# Calpain‐2‐Mediated Endothelial Focal Adhesion Disruption in Thoracic Aortic Dissection

**DOI:** 10.1002/advs.202501112

**Published:** 2025-04-02

**Authors:** Xiaomei Teng, Yansong Wang, Haoyue Huang, Yinglong Ding, Jun Wang, Meili Liu, Kun Song, Lianbo Shao, You Yu, Ziying Yang, Zhenya Shen

**Affiliations:** ^1^ Department of Cardiovascular Surgery of the First Affiliated Hospital of Soochow University 899 Pinghai Road Suzhou 215006 China; ^2^ Institute for Cardiovascular Science Soochow University 178 Ganjiang Road Suzhou 215006 China

**Keywords:** Calpain‐2, focal adhesion, Itgav, Talin, thoracic aortic dissection

## Abstract

Thoracic aortic dissection (TAD) is a life‐threatening condition with high mortality rates. Recent research suggests a potential link between early‐stage TAD and endothelial barrier dysfunction, although the underlying mechanisms remain unclear. Single‐cell RNA sequencing data from patients reveal that dysregulated Calpain‐2 expression modulates endothelial focal adhesion proteins, serving as an early pathological hallmark and driver of TAD. Elevated plasma calpain activity is strongly associated with an increased risk of TAD and organ dysfunction. Both endogenous and exogenous calpain inhibitors effectively prevent TAD onset and progression in murine models induced by β‐aminopropionitrile (BAPN). In early TAD, endothelial junction integrity in the ascending aorta and aortic arch is compromised. Endothelial‐specific deletion of Capns1 mitigates early and sustained endothelial focal adhesion damage by reducing aberrant expression of Integrin alpha‐V(Itgav), vinculin, and talin‐1, thereby decreasing TAD incidence. In contrast, macrophage‐specific Capns1 knockout does not impact TAD development but accelerates aortic dissection rupture in later stages. Mechanistically, angiotensin II upregulates Calpain‐2, leading to endothelial focal adhesion activation through talin1 cleavage and Itgav assembly, thereby compromising endothelial integrity and permeability. These findings identify potential therapeutic targets for TAD prevention and treatment.

## Graphical abstract

The elevated expression of Capn2 in endothelial cells activates focal adhesion signaling by cleaving Talin1 and assembling Itgav, which compromises the integrity of the endothelial barrier and induces vascular inflammation. Inhibition of calpain through both pharmacological and transgenic approaches has effectively suppressed the onset and progression of TAD, thereby presenting promising new strategies for intervention.

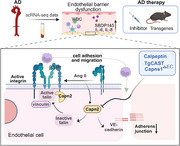

## 1 Introduction

Thoracic aortic dissection (TAD) is a critical condition that requires immediate surgical intervention.^[^ ^1^ ^]^ While patients are often asymptomatic before the onset, the disease can progress rapidly, with mortality rates reaching up to 75% within the first 72 h if left untreated.^[^ ^2^ ^]^ The absence of effective medical therapies for aortic dissection is primarily due to an incomplete understanding of the mechanisms driving disease progression.

Single‐cell RNA sequencing (scRNA‐seq) data from tissues of patients with TAD have identified key cell types in aortic tissue, including vascular smooth muscle cells (VSMCs), fibroblasts (FBs), endothelial cells (ECs), and immune cells such as monocytes/macrophages, T lymphocytes, and B lymphocytes. Notably, TAD tissues exhibit a reduction in non‐immune cells alongside a marked increase in immune cell populations compared to controls.^[^ ^3^, ^4^ ^]^

However, most data on cell populations come from patients with advanced disease requiring surgical intervention, limiting insight into early cellular changes during TAD onset and progression. scRNA‐seq analysis of murine models across early to late TAD stages reveals that early TAD is characterized by gene alterations affecting endothelial tight junctions, which disrupt barrier function and increase permeability.^[^ ^5^ ^]^ Inflammatory cell infiltration intensifies as the disease progresses.^[^ ^6^, ^7^ ^]^ Therefore, a detailed understanding of the specific patterns of endothelial barrier damage in TAD would significantly enhance knowledge of disease evolution.

Endothelial integrity and permeability are tightly regulated by cell adhesion structures.^[^ ^8^ ^]^ Focal adhesions (FAs), protein complexes that anchor cells to the extracellular matrix,^[^ ^9^ ^]^ are critical for maintaining aortic structural integrity and function.^[^ ^10^ ^]^ These complexes consist of various signaling proteins, catalytic enzymes, and cytoskeletal components, including integrins, talin, vinculin, and calpain.^[^ ^11^, ^12^ ^]^ FAs are implicated in numerous biological processes, such as endothelial cell branching during angiogenesis,^[^ ^13^ ^]^ as well as in skeletal diseases,^[^ ^14^ ^]^ diabetic retinopathy,^[^ ^8^ ^]^ and breast cancer.^[^ ^15^ ^]^ The focal adhesion scaffold gene TES was identified as a novel causal gene for thoracic aortic aneurysm (TAA) by modulating VSMC phenotypes.^[^ ^10^ ^]^ However, the role of focal adhesion dysfunction in TAD progression remains unclear.

This study analyzed scRNA‐seq data from human aortic tissue with and without TAD. Investigation of intercellular communication and bulk RNA sequencing data from BAPN‐induced TAD mouse models identified Calpain‐2 as a key driver of the focal adhesion pathway, essential for TAD onset and progression. Findings revealed a Capn2–talin–Itgav axis contributing to endothelial adhesion defects. Moreover, Calpain inhibition reduced TAD development and improved survival rates in the mouse model. These insights provide a new understanding of the mechanisms driving TAD initiation and progression, highlighting potential therapeutic targets for human TAD treatment.

## 2 Results

### 2.1 Aberrant Calpain‐2 Expression in the Aortas of Patients with TAD

**Figure 1:**
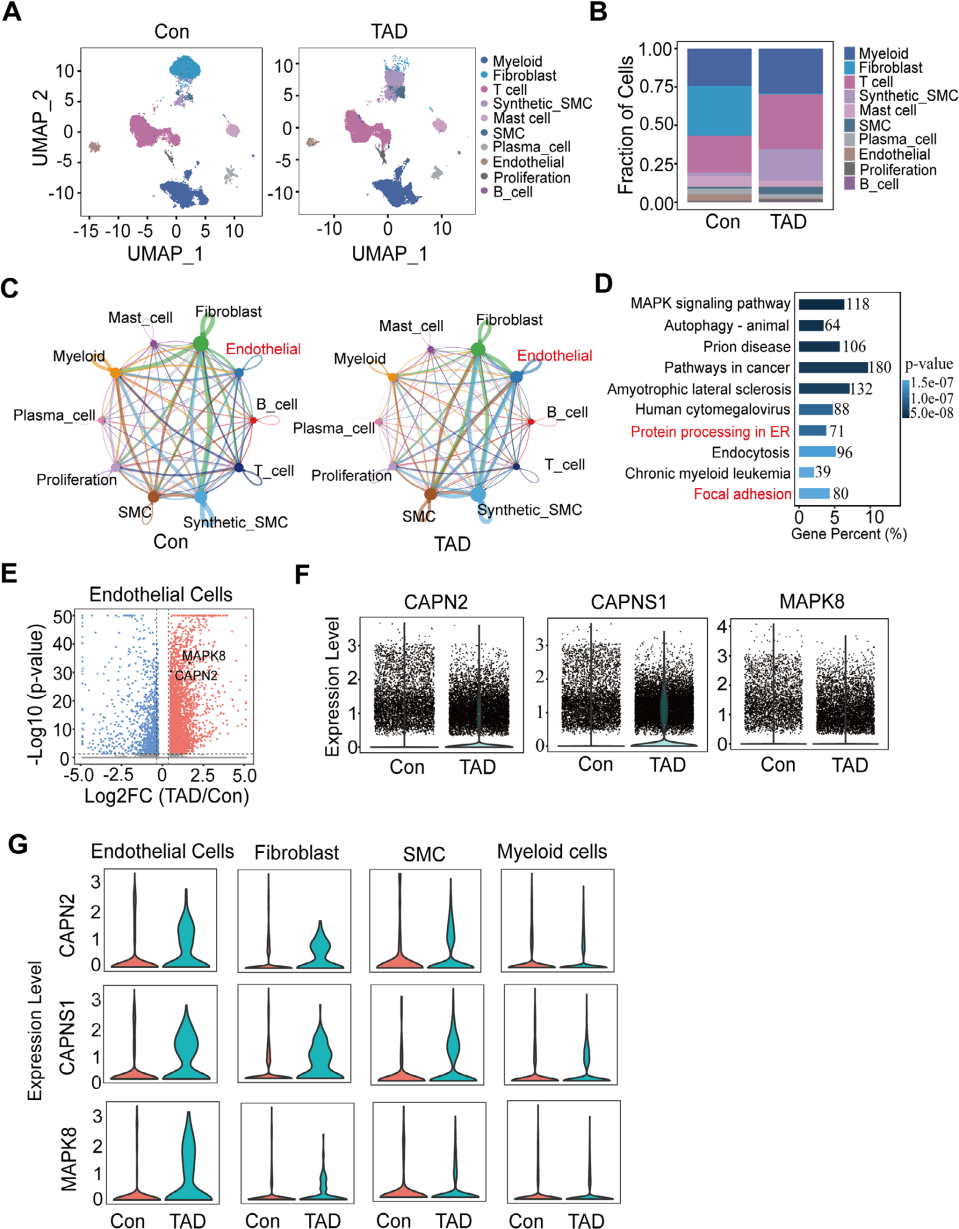
General analysis and annotation of cell clusters from both normal aorta and aortic dissection. A) UMAP representation of the control (Con) and thoracic aortic dissection (TAD) groups. *n* = 3 pregroup. B) Proportions of various cell types present in either aortic dissection or normal aorta. C) Network diagram illustrating the weight and number of receptor–ligand interactions among the primary cells in the Con and TAD groups. D) Kyoto Encyclopedia of Genes and Genomes (KEGG) pathway analysis of the differentially expressed genes (DEGs) in the endothelial cell cluster between TAD patients and controls. E) Volcano plot depicting differential gene expression in endothelial cells, comparing the TAD group to the normal group. F and G) Violin plot showing the expression levels of CAPN2, CAPNS1, and MAPK8 in single cells from TAD patients and controls (F), as well as in selected cell types (G).

After filtering, a total of 48 020 cells were included in the analysis of the single‐cell RNA sequencing dataset (GSE222318). The UMAP plot revealed ten distinct clusters (**Figure** **1**A), with identified marker genes for these cell populations (Figure S1A, Supporting Information), and demonstrated a clear separation between cells from TAD and control cells. Notably, immune cells, specifically T and B lymphocytes, represented 36.34% of all TAD cells, highlighting the significant role of lymphocyte infiltration in TAD pathogenesis. In contrast, endothelial and fibroblast cells were markedly reduced, accounting for only 0.94% and 0.65% of the total, respectively (Figure 1B and Table S1, Supporting Information).

Cell‐cell interactions are pivotal in both normal physiological processes and disease progression. Endothelial cell interactions with other major cell types were significantly stronger in TAD tissues compared to normal tissues (Figure 1C; Figure S1B, Supporting Information). KEGG enrichment analysis of differentially expressed genes (DEGs) in endothelial cells from TAD and control groups revealed “protein processing in the endoplasmic reticulum” and “focal adhesion” as the top enriched biological processes, with MAPK8 and CAPN2 identified as key genes involved (Figure 1D; Table S2 and Figure S1C, Supporting Information). The volcano plot further validated the specificity of these gene expressions (Figure 1E).

The expression levels of *CAPN2*, *MAPK8*, and *CAPNS1*—an essential regulatory subunit that stabilizes and activates Calpain‐2—were examined in single cells and across different cell types from TAD and control groups. *CAPN2* and *CAPNS1*, but not *MAPK8*, exhibited strong induction in TAD samples (Figure 1F). A similar increase in expression was observed in endothelial cells, fibroblasts, smooth muscle cells (SMCs), and proliferating cells, while only minimal changes were noted in T cells, B cells, and mast cells (Figure 1G; Figure S1D, Supporting Information). These results suggest that Calpain‐2 likely plays a pivotal role in endothelial cell activation and intercellular communication during TAD progression.

### 2.2 Correlation Between Calpain‐2 Expression and Clinical Characteristics of Patients with TAD

**Figure 2:**
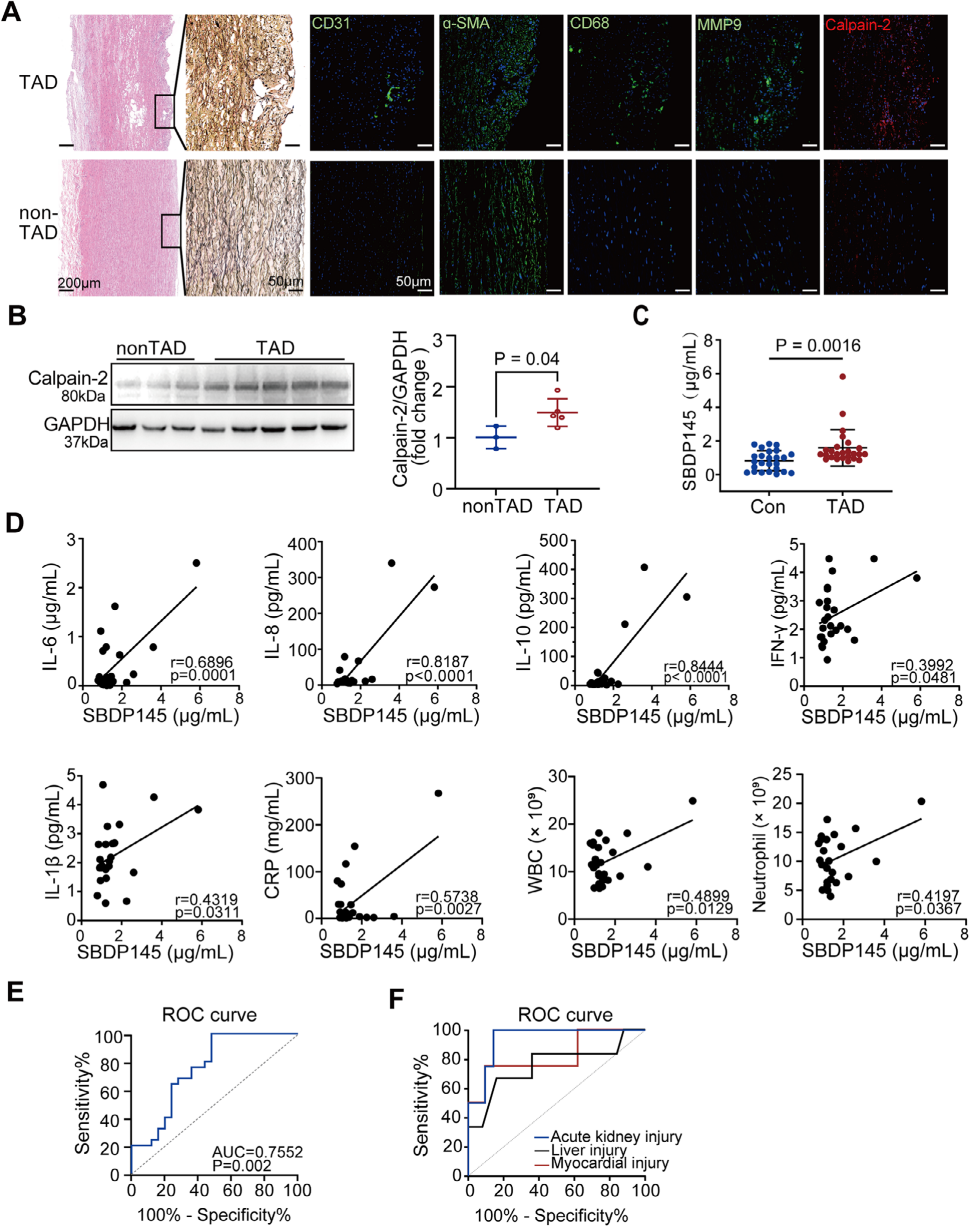
Characterization of the human thoracic aorta. A) Hematoxylin and eosin (HE), elastic van Gieson (EVG), and immunofluorescence staining(IF)techniques were employed to examine the differences in cellular structure between aorta sections from participants with TAD and those without TAD. The HE image provides an overview of the entire aortic wall tissue (scale bar, 200 µm). The EVG and IF images present magnified views of the intima of the aortic wall (scale bar, 50 µm). B) Representative Western blotting and quantification of calpain‐2 expression in aortic specimens from non‐TAD (*n* = 3) and patients with TAD (*n* = 5). Data were presented as the mean ± SD, and analyzed by unpaired two‐tailed Student's *t*‐test. C) Serum SBDP145 concentration in control and TAD patients. *n* = 25 pregroup. Data were presented as the mean ± SD and analyzed by the unpaired two‐tailed Student's *t*‐test. D) Correlation between SBDP145 concentration and various biomarkers by Pearson correlation analysis. E) ROC curve analysis of serum SBDP145 for its diagnostic value in TAD. (F) ROC curve analysis of serum SBDP145 concerning the incidence of organ dysfunction. Abbreviations: ROC, receiver operating characteristic; AUC, area under the curve; TAD, thoracic aortic dissection.

To investigate structural changes in the aortic tissue of patients with TAD, pathological features and predominant cell types within the arterial wall were assessed using HE staining, elastin van Gieson (EVG) staining, and immunofluorescence (IF). Histological analysis revealed a preserved wall structure composed of continuous adventitia, media, and intima layers. The media exhibited an uninterrupted network of elastic fibers and SMCs; however, TAD tissues displayed substantial fragmentation of elastic fibers and disruption of lamellar architecture. CD31^+^ ECs were predominantly located in the endothelium, though their quantity was markedly reduced. Additionally, the α‐SMA^+^ SMCs showed disorganized arrangement, accompanied by an increase in CD68^+^ macrophages and a notable presence of MMP9‐positive cells, particularly within the dissected aorta (**Figure** **2**A). Calpain‐2 levels were significantly higher in TAD tissues compared to non‐TAD aortic samples (Figure 2B).

Clinical and laboratory data indicated that patients with TAD were predominantly older men, exhibiting elevated leukocyte, neutrophil, and monocyte counts, alongside markedly increased C‐reactive protein (CRP) levels, reflecting an acute inflammatory response at the onset of aortic dissection onset. Furthermore, biomarkers such as D‐Dimer, ALT, AST, creatinine, cystatin C, troponin, brain natriuretic peptide, and blood glucose were elevated, suggesting multi‐organ dysfunction. Inflammatory cytokines, including IL‐6, IL‐8, and IL‐10, were also significantly elevated (Tables S3–S5, Supporting Information).

CAPN2, a member of the extensive Ca^2+^‐activated cysteine protease family, is characterized by its ability to cleave target proteins. Specifically, calpain processes αII‐spectrin into 145 kDa breakdown products (SBDP145), which serve as a specific substrate indicative of calpain activity.^[^ ^16^ ^]^ To examine the relationship between Calpain‐2 and the pathophysiological alterations in patients with TAD, serum SBDP145 concentrations were measured using an ELISA kit. The TAD group demonstrated significantly elevated SBDP145 levels compared to healthy controls (Figure 2C and Table S6, Supporting Information). Spearman's correlation analysis revealed positive correlations between serum SBDP145 levels and inflammatory markers (IL‐6, IL‐8, IL‐10, TNF‐α, and IL‐1β), as well as with WBC counts, CRP levels, and neutrophil counts. No significant correlations were observed with other parameters (Figure 2D). Receiver operating characteristic (ROC) analysis further confirmed that serum SBDP145 has diagnostic potential for TAD (Figure 2E).

Acute thoracic aortic dissection frequently results in varying degrees of organ dysfunction, with myocardial, renal, and hepatic injuries defined by established criteria.^[^ ^17^, ^18^ ^]^ Patients were stratified into high and low SBDP145 groups based on the median value of 1207.10 pg mL^−1^. Results indicated that elevated Calpain activity correlated with a higher likelihood of organ dysfunction (Table S7, Supporting Information). ROC curve analysis also demonstrated that serum SBDP145 levels hold predictive value for organ dysfunction in patients with TAD. The areas under the curve (AUCs) for SBDP145 predicting myocardial, liver, and kidney dysfunction were 0.7567 (95% CI: 0.502–1.011, *p* = 0.0542), 0.8214 (95% CI: 0.556–1.087, *p* = 0.0454), and 0.9405 (95% CI: 0.844–1.037, *p* = 0.0061), respectively (Figure 2F). These findings suggest a strong association between heightened Calpain activity and organ dysfunction in TAD, particularly in the liver and kidneys.

### 2.3 Pharmacologic Blockade of Calpain Protects Mice from BAPN‐Induced TAD

To investigate the causal role of Calpain in aortic dissection, a well‐established mouse model of TAD was employed, in which BAPN was administered through drinking water, followed by treatment with the pharmacological Calpain inhibitor, Calpeptin.^[^ ^19^ ^]^ Survival rates, TAD incidence, and the integrity of elastic fibers within the aortic wall were evaluated in both male and female mice. The results revealed that BAPN‐treated male mice exhibited lower survival rates, with earlier mortality from aortic rupture, and a higher incidence of aortic aneurysm or dissection compared to females. Notably, Calpain inhibition conferred a protective effect against BAPN‐induced TAD formation and progression in both sexes (Figure S2, Supporting Information), prompting separate statistical analysis for male and female groups.

After 2 weeks of BAPN treatment, both male and female mice showed significant weight loss, occasionally accompanied by aortic aneurysms or dissections. Ultrasonography demonstrated that prolonged BAPN exposure caused substantial aortic dilation, particularly at the arcus aortae. By the third week, a significant increase in aortic diameter was observed, while no notable changes were detected in the Calpeptin‐treated groups (Figures S3 and S4 and Tables S8 and S9, Supporting Information).

**Figure 3:**
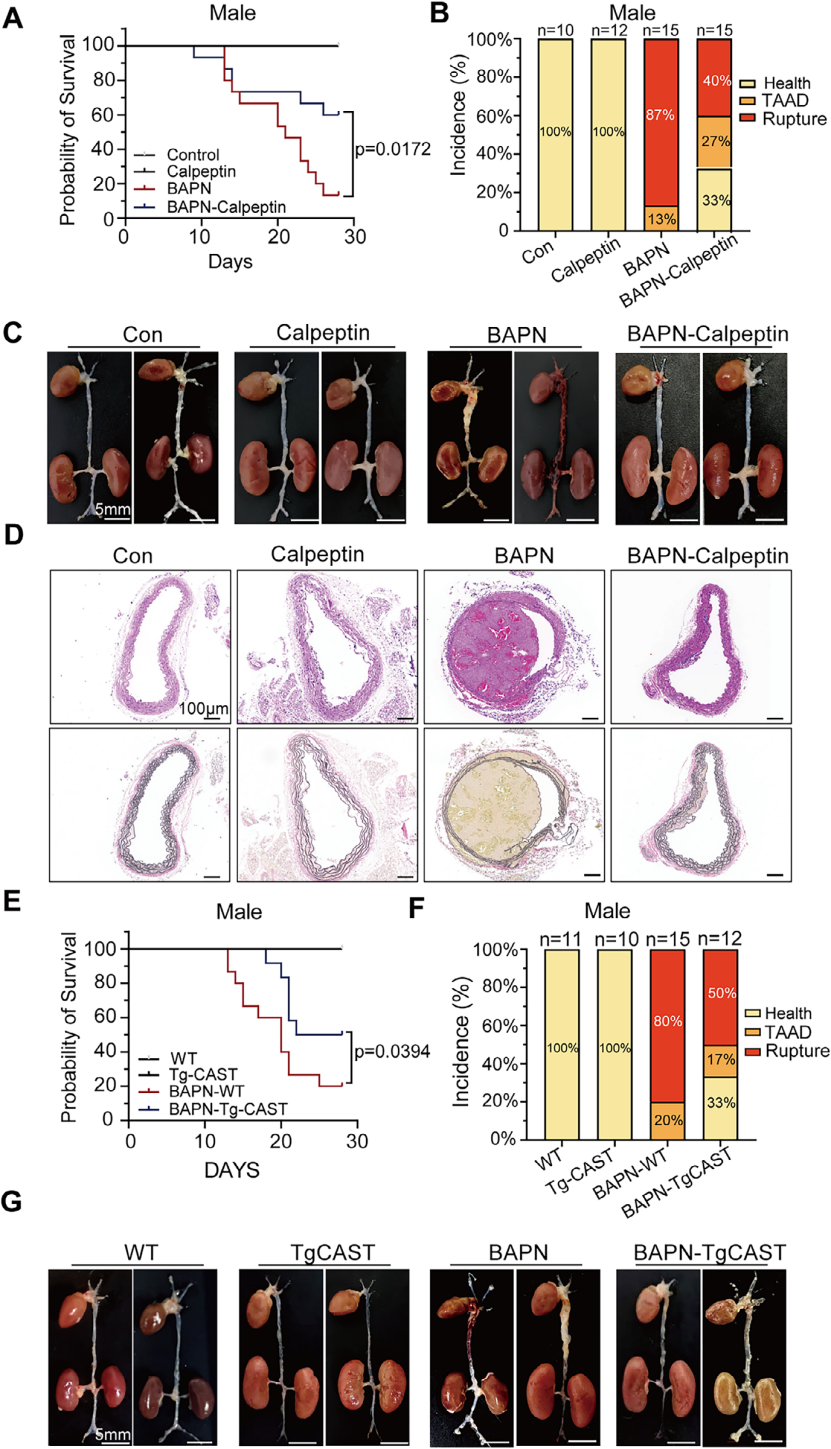
Inhibition of calpain reduced BAPN‐induced TAD formation in male mice. (A–D) Pharmacological inhibition of calpain using Calpeptin. Con, *n* = 10; Calpeptin, *n* = 12; BAPN, *n* = 15; BAPN‐Calpeptin, *n* = 15. A) The survival rate was estimated using the Kaplan–Meier method and compared via the log‐rank test. B) The incidence of TAAD. C) Representative macrographs of the aorta. scale bar: 5 mm D) Representative microscopic images of aorta sections stained with H&E and EVG (scale bar: 100 µm). (E–G) Overexpression of calpastatin (Tg‐CAST) and littermate wild‐type (WT) mice treated with BAPN for 28 days. WT, *n* = 11; Tg‐CAST, *n* = 10; BAPN, *n* = 15; BAPN‐TgCAST, *n* = 12. E) The survival rate is depicted using the Kaplan–Meier method and compared via the log‐rank test. F) The incidence of TAD. G) Representative macrographs of the aorta. scale bar: 5 mm.

Calpeptin administration significantly reduced mortality from aortic rupture and lowered the incidence of thoracic aortic aneurysm and dissection (TAAD), as well as elastin fragmentation in both male (**Figure** **3**A–D) and female mice (Figure S4, Supporting Information). These findings suggest that pharmacological inhibition of Calpain effectively mitigates TAD onset and prevents aortic rupture.

### 2.4 Endogenous Specific Inhibitor Prevents BAPN‐Induced TAD Formation

Calpain‐1 and Calpain‐2 activities are tightly regulated by calpastatin, a specific endogenous inhibitor.^[^ ^20^ ^]^ To assess the inhibitory role of calpastatin, transgenic mice overexpressing calpastatin (Tg‐CAST) and their wild‐type (WT) littermates were subjected to BAPN treatment. Throughout their lifespan, Tg‐CAST mice exhibited no apparent global phenotype, and their aortic anatomy remained similar to that of WT mice under both normal and BAPN‐treated conditions. As expected, calpastatin overexpression significantly reduced mortality associated with aortic rupture. Specifically, during the 4‐week treatment period, 12 out of 15 WT mice and 6 out of 12 Tg‐CAST mice treated with BAPN succumbed to aortic rupture, while no mortality occurred in either WT or Tg‐CAST male mice receiving vehicle treatment (Figure 3E–G and Tables S10 and S11, Supporting Information). Similar protective effects were observed in Tg‐CAST female mice, further supporting the causal role of Calpain in TAD onset and aortic rupture (Figure S5, Supporting Information).

### 2.5 Transcriptome Profiling Reveals Calpain Inhibition Effects on TAD Progression

**Figure 4:**
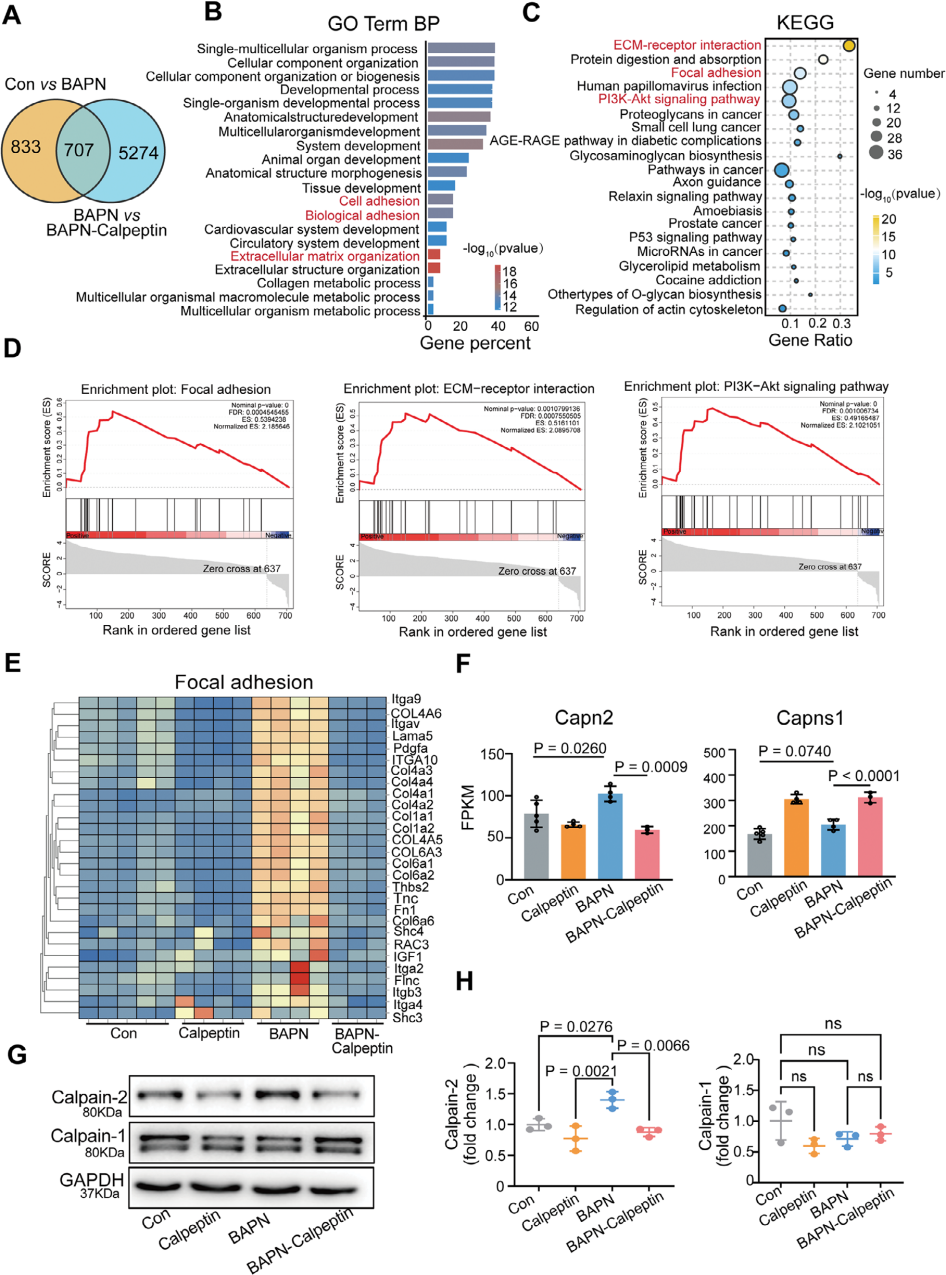
RNA‐seq analysis for pharmacological inhibition of Calpain. A) A Venn diagram analysis. B) Biological Process (BP) of Gene Ontology (GO) term analysis for 707 genes. C) KEGG analysis for 707 genes. D) Gene Set Enrichment Analysis (GSEA) for 707 genes. E) A heatmap depicting the focal adhesion pathway. F) FPKM values for Capns and Capns1 across four groups. Con, *n* = 5; Calpeptin, *n* = 4; BAPN, *n* = 4; BAPN‐Calpeptin, *n* = 3. G) Representative Western blot results for Calpain‐1 and Calpain‐2. H) Quantification of calpain‐2 expression. *n* = 3 per group. Data are presented as the mean ± SD. *p*‐values were calculated by One‐way ANOVA, followed by Tukey's multiple comparision.

To elucidate transcriptomic changes associated with Calpain inhibition, mRNA sequencing was conducted on mice treated with BAPN, both with and without Calpeptin. The analysis revealed that 1381 genes were upregulated in the BAPN samples (Figure S6A, Supporting Information), while 2013 genes were downregulated in the BAPN‐Calpeptin group (Figure S6B, Supporting Information). These DEGs were involved in regulating the PI3K‐Akt signaling pathway, focal adhesion, and ECM‐receptor interactions (Figure S6C,D, Supporting Information). A Venn diagram highlighted DEGs specifically associated with Calpain inhibition (**Figure** **4**A), as detailed in Table S13 (Supporting Information). Gene ontology (GO) analysis of 707 genes identified terms related to cell adhesion, biological adhesion, and extracellular matrix organization (Figure 4B). KEGG and GSEA pathway analyses of the same gene set revealed that ECM‐receptor interaction, focal adhesion, and the PI3K‐Akt signaling pathway were the most significantly downregulated pathways in the BAPN‐Calpeptin samples (Figure 4C,D). Integration of these findings with human single‐cell RNA sequencing data (Figure 1D) further confirmed that focal adhesion plays a pivotal role in TAD progression (Figure 4E).

To investigate the involvement of specific genes within the Calpain family, the expression levels of 15 family members were examined across four sequencing datasets. The results demonstrated significant upregulation of Capn2, Capn3, Capn6, Capn7, and Capns1 in the BAPN‐treated group, with reduced expression following Calpain inhibition (Figure S6E–G, Supporting Information). While Calpain‐1 and Calpain‐2 are ubiquitously expressed, Capns1 acts as the regulatory subunit for both enzymes. Other Calpain family members exhibit tissue‐specific expression. Notably, Capn2 emerged as the most abundantly expressed differentially regulated gene in this RNA‐seq dataset. FPKM values and Western blot analysis further confirmed that Calpain‐2, rather than Calpain‐1, plays a critical role in TAD onset and progression (Figure 4F–H).

### 2.6 Identification of Common Genes in Focal Adhesion Pathway in BAPN‐Induced Aortic Tissue

**Figure 5:**
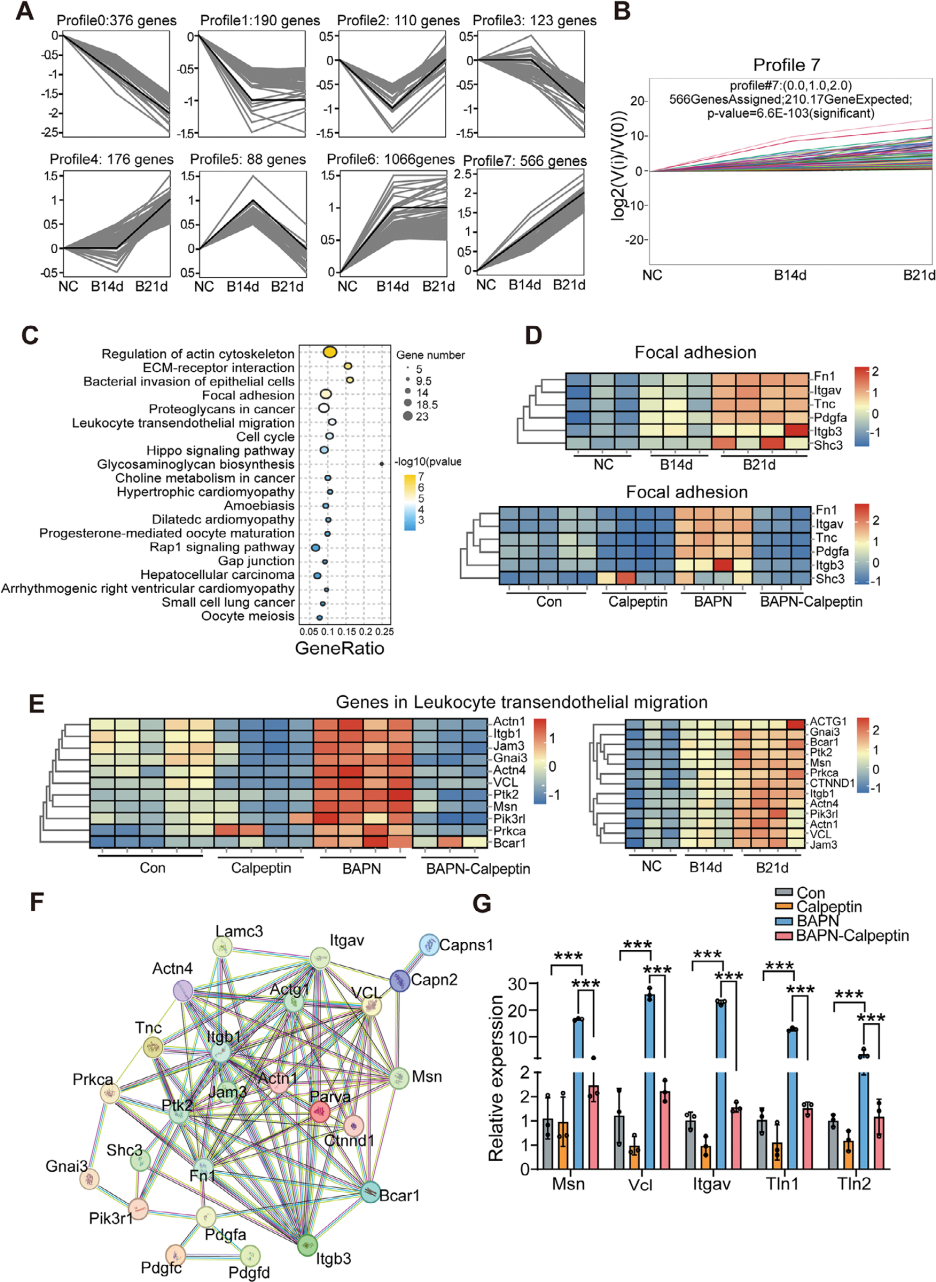
Time‐series RNA‐seq analysis of the thoracic aorta in mice. A) A Trend analysis (Series Test of Cluster) for the trend characteristics of gene abundance across different groups. B) The expression trend of genes in Profile 7. C) Top 20 enrichment analysis results for KEGG pathway in Profile 7. D) Heatmap depicting genes associated with the focal adhesion pathway. E) Heatmap of genes involved in leukocyte transendothelial migration. F) Protein‐protein interaction (PPI) network between the focal adhesion and leukocyte transendothelial migration pathways. The nodes represent proteins and edges denote interactions between pairs of proteins. G) qPCR results from aortic tissue confirmed the expression levels of Itgav, Msn, Vcl, TIn1, and TIn2. *n* = 3 per group. Statistical significances were determined by two‐way ANOVA with Tukey's multiple comparison. ***p < 0.001.

To investigate the in vivo mechanisms driving TAD progression, RNA sequencing of the ascending aorta and aortic arch was performed at three‐time points after BAPN administration: 0, 14, and 21 days. DEGs were identified among these groups (Figure S7A, Supporting Information). Trend analysis using the Series Test of Cluster revealed eight distinct gene expression profiles across the gradient‐injury samples (**Figure** **5**A). Functional enrichment analysis was conducted on genes in profile 0, which showed continuous decreases in 376 genes, and profile 7, which exhibited increases in 566 genes (Figure S7B, Supporting Information; Figure 5B). Genes in profile 0 were primarily associated with various metabolic pathways, while those in profile 7 were linked to critical pathways, including ECM‐receptor interaction, focal adhesion, and leukocyte transendothelial migration (Figure S7C,D, Supporting Information; Figure 5C). This suggests that Calpain‐mediated regulation of focal adhesion plays a key role in TAD progression. Several genes involved in the focal adhesion pathway were identified by intersecting both RNA‐seq datasets, including Fn1, Pdgfa, Tnc, Itgav, Shc3, and Itgb3 (Figure S7E, Supporting Information).

Heatmap analysis demonstrated a progressive increase in the expression of these genes with prolonged BAPN treatment, with a notable decrease in their expression following Calpain inhibition (Figure 5D). A similar expression pattern was observed for genes related to leukocyte transendothelial migration (Figure 5E). Moreover, as BAPN‐induced aortic injury worsened, CAPN2 mRNA expression steadily increased (Figure S7F, Supporting Information).

PPI analysis of these two signaling pathways revealed that Calpain‐2 primarily regulates the functions of integrin ɑv (Itgav), vinculin (Vcl), and moesin (Msn), all of which are essential for focal adhesion and leukocyte transendothelial migration (Figure 5F). Quantitative PCR analysis of aortic tissue further confirmed that Itgav, Msn, Vcl, and Talin1—a key structural component of focal adhesions—were elevated in BAPN‐induced aortic tissue, with their expression significantly decreased following Calpain inhibition (Figure 5G). In aortic tissue samples from patients with TAD, it was also confirmed that the expression of Itgav, Itgb3, and Talin1 was significantly increased compared to non‐TAD aortic tissue (Figure S8, Supporting Information). These results suggest that Calpain‐2 plays a pivotal role in TAD progression by modulating focal adhesion and leukocyte transendothelial migration in endothelial cells.

### 2.7 The Deletion of Endothelial Capns1 Prevents the Formation and Progression of TAAD by Regulating Focal Adhesion

**Figure 6:**
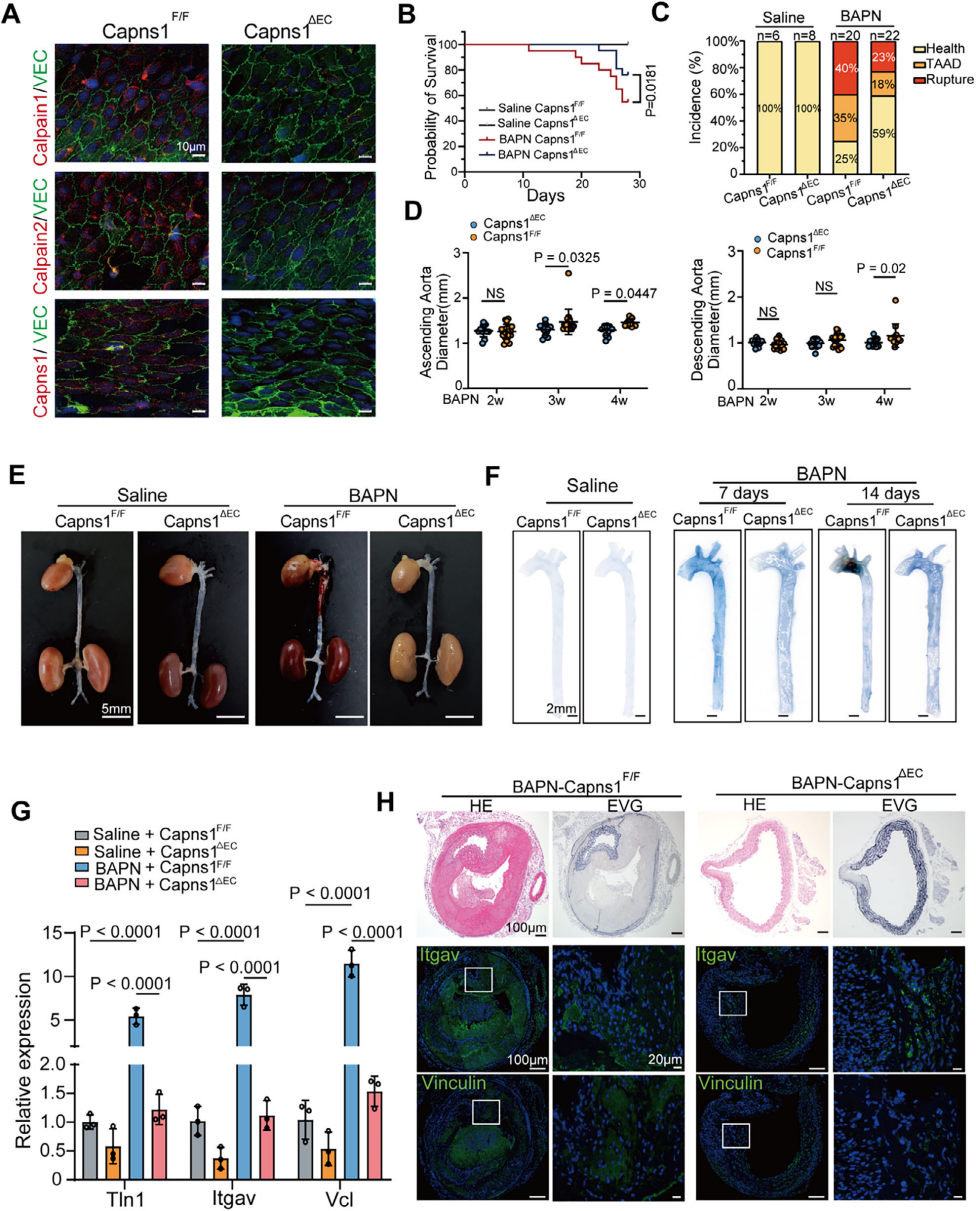
Conditional knockout of capns1 in endothelial cells prevented the formation of BAPN‐induced TAAD. A) En‐face immunofluorescence staining images depicting Calpain‐1, Calpain‐2, Capns1, and endothelial marker VE‐cadherin (VEC) in the descending aorta from Capns1^F/F^ and Capns1^△EC^ mice. Nuclei were counter‐stained using DAPI. Scale bar, 10 µm. B) The survival rate was estimated using the Kaplan‐Meier method and compared via the log‐rank test. C) The incidence of TAAD in Capns1^F/F^ and Capns1^△EC^ mice following 4 weeks of BAPN treatment. Saline‐Capns1^F/F^, *n* = 6; Saline‐Capns1^△EC^, *n* = 8; BAPN‐Capns1^F/F^, *n* = 20; BAPN‐Capns1^△EC^, *n* = 22. D) Quantification of the maximal diameters of both the ascending and descending aorta over the 4‐week period post‐BAPN treatment. Statistical significances were determined by two‐way ANOVA with Sidak's multiple comparision. E) Gross images depict the morphology of the entire aorta. Scale bar: 5 mm. F) The permeation of Evans blue dye into the thoracic aortas of Capns1^F/F^ and Capns1^△EC^ mice as assessed at 7 and 14 days after BAPN administration. G) Quantitative data on mRNA expression levels for Itgav, Vcl, and TIn1 (*n* = 3 per group). Statistical significances were determined by two‐way ANOVA with Tukey's multiple comparision. H) Hematoxylin and eosin(HE), elastic van Gieson (EVG), and immunofluorescence (IF) staining of Itgav and Vinculin in cross‐sections of the Capns1^F/F^ and Capns1^△EC^ aorta.

To elucidate the role of endothelial Calpain in the development of TAAD, endothelial‐specific Capns1 knockout (Capns1^ΔEC^) mice were generated, as previously described.^[^ ^21^ ^]^ In these mice, the expression levels of Calpain‐1 and Calpain‐2 in endothelial cells were significantly reduced (**Figure** **6**A). Since no sex‐based differences were observed in the protective effects of Calpain inhibition, male mice were used for this experiment. Calpain activity was activated in the BAPN‐induced TAD model mice, and it was significantly reduced following the deletion of Calpain in endothelial cell (Figure S9A, Supporting Information). Capns1^ΔEC^ mice exhibited significantly reduced mortality rates and lower incidences of TAAD compared to Capns1^F/F^ mice after BAPN induction. Furthermore, dilation of both the ascending and descending aorta was effectively prevented in Capns1^ΔEC^ mice (Figure 6B–E).

The vascular endothelium serves as a regulated barrier, with endothelial integrity and permeability being crucial determinants.^[^ ^7^ ^]^ To assess whether endothelial barrier function is disrupted during early TAAD formation, a vascular permeability assay was conducted at 7 and 14 days post‐BAPN administration. In Capns1^F/F^ mice treated with BAPN for 7 days, Evans blue dye permeated the vascular layers of the thoracic aorta, including the ascending aorta, arch, and descending segments. Vascular leakage became more pronounced by day 14, particularly at the aortic arch (Figure 6F).

These results suggest that the integrity of endothelial junctions in the ascending aorta and aortic arch is compromised during the early stages of TAAD, leading to earlier dilation of the ascending aorta compared to the descending aorta. The integrity and permeability of the endothelium are tightly regulated by adhesion junctions and tight junction molecules.^[^ ^8^ ^]^ Notably, the abnormal overexpression of focal adhesion components, including Itgav, Vinculin, and Talin‐1, was significantly attenuated in Capns1ΔEC mice compared to Capns1F/F mice (Figure 6G,H), which directly reduced inflammatory factors (IL‐6 and TNF‐ɑ) and the infiltration of CD68^+^ macrophages (Figure S9B, Supporting Information).

The role of inflammatory cells, particularly macrophages, has drawn considerable attention in the aortic aneurysm and dissection research. In this study, we utilized a mouse model of TAAD with a macrophage‐specific knockout of Capns1 was employed to investigate the impact of macrophages. Our findings indicate that while macrophages do not influence the early formation and progression of TAD, they play a role in promoting the rupture of aortic dissection at later stages (Figure S10, Supporting Information).

### 2.8 Calpain‐2 Regulation of Integrin Activation and Talin‐Cleavage in Endothelial Cells

**Figure 7:**
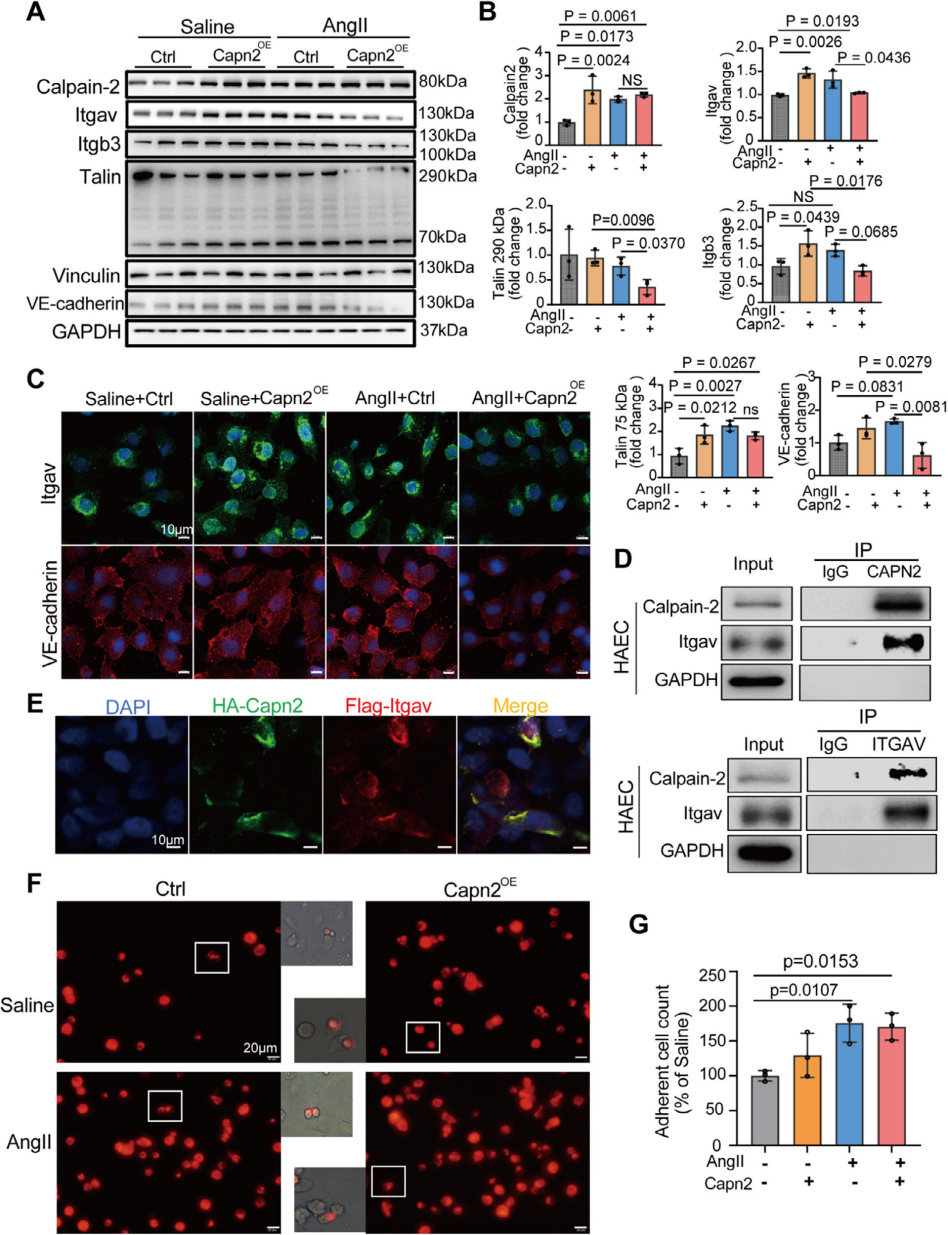
The regulation of integrin activation and talin cleavage by Calpain‐2 in endothelial cells. Human aortic endothelial cells (HAECs) were transduced with Calpain‐2 via lentiviral transfection and subsequently treated with 1 µmol Angiotensin II (Ang II) for 24 h. A) The expression levels of Calpain‐2, Itgav, Itgb3, Talin, Vinculin, and VE‐cadherin were assessed using Western blotting. Representative Western blot images were presented, with GAPDH serving as the loading control. B) Quantitative data from the Western blot analysis (*n* = 3 per group). C) Representative images of immunofluorescence staining for Itgav and VE‐cadherin in HAECs. Scale bar: 10 µm. D) Cell lysates from HAECs underwent co‐immunoprecipitation (Co‐IP) using anti‐Calpain‐2 or anti‐Itgav antibodies, followed by Western blotting. E) HA‐Capn2 or Flag‐Itgav were co‐transfected into HEK293T cells, demonstrating the colocalization of Calpain‐2 (green) and Itgav (red) in these cells. Scale bar:10 um. F) An adhesion assay was performed to evaluate the adhesion of THP‐1 monocytes to HAECs. Representative images showing DiI‐labeled THP‐1 monocyte adhesion to HAECs. Scale bar: 20 um. G) The relative number of THP‐1 monocytes adhering to HAECs following exposure to 1 µm Ang II for 24 h was presented. Statistical significances were determined by One‐way ANOVA with Tukey's multiple comparision.

Focal adhesions are a critical role for regulating the endothelial barrier and maintaining its integrity.^[^ ^22^ ^]^ These multiprotein complexes, primarily composed of integrins and intracellular cytoskeletal proteins such as integrin, vinculin, and talin‐1,^[^ ^23^ ^]^ play essential roles in stabilizing and localizing VE‐cadherin at cell–cell junctions.^[^ ^22^, ^24^ ^]^ In vitro experiments with human aortic endothelial cells (HAECs) treated with 1 µmol Ang II for 24 h revealed that Ang II infusion increased the levels of Calpain‐2 and Calpain activity. Calpain‐2 overexpression, achieved via lentiviral transfection, resulted in elevated levels of Itgav, Itgβ3, and cleaved talin‐1 fragments. Notably, extensive cleavage of full‐length talin‐1 occurred, and the expression of Itgav and Itgβ3 was significantly reduced in endothelial cells overexpressing CAPN2 upon additional Ang II treatment. Furthermore, VE‐cadherin expression decreased following increased Calpain‐2 levels, while vinculin expression remained unchanged across the groups (**Figure** **7**A,B; Figure S11A,B, Supporting Information).

Immunofluorescence analysis confirmed the increased expression of adhesion mediators Itgav (green) and VE‐cadherin (red) in response to Ang II, which diminished upon overexpression of Capn2 (Figure 7C). Consistent with this, the Calpain inhibitor Calpeptin significantly reversed the downregulation of endothelial barrier mediators following Ang II treatment (Figure S11C, Supporting Information).

Although limited direct evidence links Capn2 to Itgav, co‐immunoprecipitation (Co‐IP) assays revealed a physical interaction between endogenous Capn2 and Itgav in HAECs (Figure 7D). Immunofluorescence experiments in HEK293T cells transfected with Capn2 and Itgav plasmids further confirmed that Calpain‐2 directly binds to Itgav (Figure 7E).

Adhesion assays assessed the effects of Calpain‐2 on THP‐1 monocyte adhesion to HAECs. The number of adhering THP‐1 cells significantly increased in the presence of 1 µmol Ang II; however, Capn2 overexpression did not further enhance cell adhesion (Figure 7F).

In summary, these findings suggest that focal adhesion operates through a precise feedback inhibition loop. In response to mild endothelial injury induced by Ang II or Capn2 overexpression, Calpain‐2 activates Itgav while cleaving talin‐1, promoting the interaction between the talin fragments and integrin αV and β3 subunits, thereby activating integrin. The increased expression of VE‐cadherin suggests endothelial activation. Conversely, in the presence of both Ang II treatment and Capn2 overexpression, moderate endothelial damage results in extensive talin‐1 cleavage, reduced Itgav activation, decreased VE‐cadherin expression, and weakened cell connections. These findings indicate that Calpain‐2 facilitates endothelial barrier dysfunction through an Itgav/talin cleavage mechanism.

## 3 Discussion

The aortic wall consists of dynamic cell populations that undergo substantial changes during compression, injury, repair, and remodeling processes.^[^ ^25^ ^]^ Most current models of TAD pathogenesis have focused on the phenotypic switching of VSMCs as a central event driving degenerative responses.^[^ ^26^ ^]^ However, this study is the first to identify aberrant endothelial focal adhesion proteins as an early pathological hallmark of TAD. By integrating clinical informatics with experimental validation, the study highlights the critical role of the Calpain2–Talin1–Itgav axis in maintaining endothelial adhesion and junction integrity. Targeting Calpain2 was shown to significantly reduce both the onset and rupture of TAD, offering promising insights for early prediction and treatment of the disease.

Previous scRNA‐seq studies have largely focused on samples from the ascending aorta of patients with acute thoracic aortic dissection, aiming to uncover the molecular and cellular mechanisms underlying the disease.^[^ ^27^ ^]^ These studies consistently observed a significant reduction in SMCs and ECs, coupled with an increase in macrophages within the TAD‐affected aorta. Conversely, fibroblast populations remained largely unchanged or even decreased.^[^ ^3^, ^27^ ^]^ Recent research has mapped an scRNA‐seq atlas of the human dissected ascending aorta across acute, subacute, and chronic stages of TAD.^[^ ^4^ ^]^ The acute and subacute phases revealed a similar pattern of phenotypic transitions in SMCs, distinct from the chronic phase. The subacute stage appears to be the optimal window for guiding aortic remodeling toward positive outcomes.^[^ ^28^ ^]^ Thus, sequencing data from subacute phases could help in designing more precise therapies to prevent aortic dissection progression.

In this study, reanalysis of sequencing data from the subacute groups revealed an increased immune cell population and a decreased presence of ECs and fibroblasts. Interactions between ECs and other major cell types were significantly stronger in TAD tissue, with focal adhesion emerging as the primary mechanism mediating cell–cell communication. A previous study on endothelial clusters demonstrated that endothelial tight junction disruption occurs early in TAD progression.^[^ ^5^ ^]^ Additionally, another study identified the focal adhesion pathway as the most enriched functional cluster associated with early endothelial barrier dysfunction in abdominal aortic aneurysm.^[^ ^7^ ^]^ Moreover, bulk sequencing data from BAPN‐induced TAD mice at three‐time points, combined with Calpain inhibitor treatment, as well as studies using CoQ10 or TPA in TAD models,^[^ ^4^ ^]^ consistently confirmed that focal adhesion plays a pivotal role in the onset and progression of TAD.

Emerging evidence highlights the pivotal role of the calpain proteolytic system in degenerative vascular disorders.^[^ ^29^, ^30^ ^]^ Calpains, a family of 15 calcium‐dependent cysteine proteases in mammals, include the two conventional isoforms, calpain‐1 and calpain‐2, which are widely expressed. These isoforms form heterodimers with a common regulatory subunit, CAPNS1, essential for their catalytic activity.^[^ ^31^ ^]^ While calpain‐1 and calpain‐2 are predominantly present across most vascular systems, other isoforms exhibit tissue specificity and have been less studied in vascular regulation due to their low expression in the healthy aorta.^[^ ^30^ ^]^ The present study demonstrates a significant elevation of calpain‐2, but not other isoforms, in both human TAD samples and mouse AD models. While calpain‐1 and calpain‐2 are predominantly present across most vascular systems, other isoforms exhibit tissue specificity and have been less studied in vascular regulation due to their low expression in the healthy aorta.^[^ ^30^ ^]^ The present study demonstrates a significant elevation of calpain‐2, but not other isoforms, in both human TAD samples and mouse AD models.

Prior studies have shown that pharmacological inhibition of calpain reduces the incidence of angiotensin II (AngII)‐induced abdominal aortic aneurysms (AAAs) in mice.^[^ ^32^ ^]^ However, calpain‐1 deficiency does not affect the formation of ascending or abdominal aneurysms, as compensatory upregulation of calpain‐2 restores overall calpain activity.^[^ ^33^ ^]^ Moreover, inducible whole‐body depletion of calpain‐2, along with its deletion in fibroblasts and smooth muscle cells (FMSC), significantly reduces AngII‐induced AAAs.^[^ ^34^ ^]^ In contrast, calpain‐2 deficiency in leukocytes or adipocytes does not influence AAA development,^[^ ^34^, ^35^ ^]^ underscoring the cell‐specific role of calpain‐2 in aortic aneurysm pathogenesis. These findings suggest that calpain‐2, rather than calpain‐1, plays a critical role in aortic aneurysm development, with its effects on cellular function modulated by cell type and environmental conditions.

This study is the first to report the aberrant expression of calpain‐2 in endothelial cells, which mediates the regulation of focal adhesion proteins in both human and experimental TAD. In the early stages of the BAPN‐induced aortic dissection model, endothelial junction integrity in the ascending aorta and aortic arch was compromised. As BAPN‐induced aortic injury progressed, CAPN2 mRNA expression increased, with more pronounced leakage, especially at the aortic arch. Endothelial‐specific deletion of Capns1 reduced the overexpression of Itgav, vinculin, and talin‐1, thereby preserving endothelial barrier function and preventing the onset and rupture of TAD in mice.

Focal adhesion, a dynamic protein complex, is essential for maintaining endothelial integrity.^[^ ^8^, ^11^ ^]^ The integrin‐talin axis acts as a key signaling hub in processes such as barrier disruption and recovery.^[^ ^36^ ^]^ Our research, through sequencing analysis and experimental validation, demonstrates that AngII enhances endothelial cell adhesion and junction degradation in a calpain‐2‐dependent manner. The underlying mechanisms can be summarized as follows: 1) Calpain‐2 is activated by BAPN‐induced injury or AngII, regulating downstream integrins αv (ITGAV) and β3 (ITGb3), which are pivotal mediators of cell‐cell adhesion; 2) Calpain‐2 activation induces talin‐1 cleavage, separating its head and tail domains, which facilitates talin‐integrin binding and focal adhesion formation; 3) Proteolysis of talin‐1′s rod domain by calpain drives the disassembly of talin‐integrin complexes and the degradation of VE‐cadherin at the cell membrane.

Integrins are heterodimeric transmembrane receptors composed of α‐ and β‐chains.^[^ ^37^ ^]^ Integrin αvβ3, typically expressed at low levels in quiescent endothelial cells, is upregulated during processes such as vascularization, inflammation, and tumor angiogenesis.^[^ ^38^ ^]^ It plays a critical role in mediating the adhesion of monocytes, platelets, and endothelial cells.^[^ ^39^ ^]^ Although endothelial‐specific deletion of Itgav does not result in significant vascular defects during development, it induces colitis via TGF activation.^[^ ^40^ ^]^ This study provides the first evidence that integrin αv is a downstream target of calpain‐2, activated by calpain‐2 to facilitate focal adhesion formation.

Calpain‐mediated cleavage of talin separates its head domain from the rod domain. The head domain binds to integrin tails, while the rod domain interacts with vinculin, promoting focal adhesion assembly.^[^ ^12^, ^41^ ^]^ Talin contains several calpain cleavage sites adjacent to its vinculin‐binding helices.^[^ ^42^ ^]^ Additionally, talin‐dependent integrin activation affects VE‐cadherin organization at endothelial cell‐cell junctions.^[^ ^43^ ^]^ Calpain‐2 directly cleaves VE‐cadherin, disrupting adherence junctions and increasing endothelial hyperpermeability.^[^ ^44^ ^]^ Our findings reveal that calpain‐2‐mediated proteolysis of talin‐1 destabilizes talin‐integrin complexes and degrades VE‐cadherin, underscoring the role of focal adhesion turnover in promoting endothelial hyperpermeability.

In conclusion, this study emphasizes the critical role of endothelial focal adhesion function, regulated by calpain‐2, in TAD progression. Calpain‐2 contributes to early endothelial barrier dysfunction through a Talin1–Itgav‐mediated mechanism. Furthermore, detecting calpain activity may serve as a predictive marker for TAD onset or related organ dysfunction, and targeting calpain‐2 offers a promising strategy for early monitoring and treatment of TAD.

## 4 Experimental Section

### Materials

Antibodies for α‐SMA (rabbit IgG, 14395‐1‐AP), CD31 (rabbit IgG, 11265‐1‐AP), CD68 (rabbit IgG, 28058‐1‐AP), MMP9 (rabbit IgG, 10375‐2‐AP), Calpain‐2 (rabbit IgG, 11472‐1‐AP), Calpain‐1 (rabbit IgG, 10538‐1‐AP), VE‐cadherin (mouse IgG, 66804‐1‐Ig), Itgav (rabbit IgG, 27096‐1‐AP), Itgb3 (rabbit IgG, 18309‐1‐AP), Talin‐1 (rabbit IgG, 14168‐1‐AP), and Vinculin (mouse IgG, 66305‐1‐Ig) were sourced from Proteintech, China. All secondary antibodies and additional materials were procured from MedChemExpress (New Jersey, USA), unless otherwise specified.

### Study Population

Patients included in this study were diagnosed with type A TAD and admitted to the First Affiliated Hospital of Soochow University between January 2022 and December 2022, having undergone computed tomography angiography (CTA) of the thoracic aorta. Inclusion criteria required a confirmed diagnosis of type A aortic dissection within 48 h of symptom onset, and patients were required to be over 18 years of age. Exclusion criteria included chronic liver and renal diseases, hematological disorders, autoimmune diseases, malignant tumors, or a prior history of aortic dissection. Non‐TAD tissues were sourced from the Biospecimen Bank of the same hospital. Human thoracic aortic tissue specimens and serum samples were collected during elective surgery from consenting patients (approval number: 2022‐689). Control serum samples were obtained from the Physical Examination Center, matched by age and gender during the same period. Patients with TAD often present with varying degrees of organ dysfunction. Myocardial injury was indicated by high‐sensitivity troponin levels exceeding 52 pg mL^−1^.^[^ ^17^ ^]^ Acute kidney injury (AKI) was diagnosed based on KDIGO guidelines, which define AKI by one of the following criteria:^[^ ^45^ ^]^ 1) An increase in serum creatinine (SCr) of more than 0.3 mg dL^−1^ (≥26.5 µmol L^−1^) within 48 h; 2) Presumed or confirmed renal function impairment within 7 days, where SCr rises to more than 1.5 times the baseline value; 3) Urine output below 0.5 ml kg^−1^ h^−1^) for 6 h. Acute liver injury was defined by ALT levels exceeding five times the upper limit of normal or AST levels more than three times the upper limit, along with bilirubin levels exceeding two times the upper limit.^[^ ^18^ ^]^ All study protocols and methods involving human subjects were approved by the Ethics Committee of the First Affiliated Hospital of Soochow University and were conducted in accordance with the Declaration of Helsinki.

### General Analysis of scRNA Sequencing Data Processing

Single‐cell transcriptome data from the aortic dissection dataset GSE222318 (including three aortic dissection and three normal aorta samples) were processed using 10X Genomics' Cell Ranger software (version 3.1.0). Cells with an abnormally high number of unique molecular identifiers (UMIs) (≥8000), a mitochondrial gene percentage of ≥10%, or had fewer than 500 or more than 4000 detected genes were excluded.^[^ ^46^ ^]^ DoubletFinder (v2.0.3) was used to remove doublet GEMs. The cell‐by‐gene matrices for each sample were imported into Seurat (version 3.1.1) for downstream analysis.^[^ ^47^ ^]^ Principal components enriched with low p‐value genes were identified for clustering and dimensional reduction.^[^ ^48^ ^]^ Seurat was used for differentially expressed genes analysis.

New identifiers were established as “group_cluster” or “group_cell type” for analysis.^[^ ^49^ ^]^ The hurdle model in MAST (Model‐based Analysis of Single‐cell Transcriptomics)^[^ ^50^ ^]^ was employed to identify differentially expressed genes within a group in a single cluster. A shared‐nearest neighbor (SNN) graph was constructed based on the euclidean distance in PCA space, refined the edge weights between any two cells according to the shared overlap in their local neighborhoods (Jaccard distance). The Louvain method was utilized for clustering cells to maximize modularity (with a default resolution of 0.5).^[^ ^51^ ^]^ Harmony algorithm was applied to minimize the effects of batch effect and behavioral conditions on clustering.^[^ ^52^ ^]^

Graph‐based clustering was performed using Seurat's FindAllMarkers function, which identified marker genes and DEG functions. A 2D uniform manifold approximation and projection (UMAP) plot was generated to visualize clusters. Cell‐cell communication analysis was conducted by identifying biologically relevant ligand‐receptor interactions with CellPhoneDB (version 5.0.0) (https://www.cellphonedb.org/).^[^ ^53^ ^]^ Cell communication networks were illustrated using the R packages Igraph and Circlize.^[^ ^27^ ^]^ Differential expression significance was defined as a p_value_ad j< 0.05 and |log2foldchange| >0.36. KEGG pathway enrichment analysis was performed using R based on the hypergeometric distribution.

### Animals and Experimental Model

All animal experiments adhered to the National Institutes of Health Guidelines for the Care and Use of Laboratory Animals and were approved by the Soochow University Animal Research Ethics Committee (Ethical number 202009A471). Age‐ and sex‐matched mice were randomly assigned to experimental groups and housed under a 12‐h light/dark cycle in a specific pathogen‐free facility at Soochow University.

Three‐week‐old C57BL6/J mice of both sexes, purchased from Gempharmatech Company, were administered beta‐aminopropionitrile (BAPN) at a dosage of 1 g kg^−1^ day^−1^ (Sigma–Aldrich, A3134) in drinking water for up to four weeks to induce aortic aneurysm or dissection.^[^ ^54^ ^]^ To assess the therapeutic potential of calpain inhibition in TAD, mice were randomly assigned to receive either a vehicle or the calpain inhibitor Calpeptin (0.01 mg mL^−1^ g^−1^ body weight via intraperitoneal injection, administered three times per week), starting 3 days prior to BAPN treatment.^[^ ^19^ ^]^

Transgenic mice overexpressing calpastatin (TgCAST) were generously provided by Dr. TQ Peng.^[^ ^20^ ^]^ WT and TgCAST mice were divided into four groups: WT‐sham, TgCAST‐sham, WT‐BAPN, and TgCAST‐BAPN. Endothelial‐specific Capns1 knockout (Capns1^ΔEC^) mice were generated as previously described.^[^ ^21^ ^]^ Both Capns1^ΔEC^ and their WT littermates received BAPN for four weeks. Macrophage‐specific Capns1 (balb/c) mice (Capns1^ΔLKO^) were obtained as previously described.^[^ ^55^ ^]^ Both Capns1^ΔLKO^ mice and their WT littermates underwent BAPN treatment for four weeks

Mouse weight was recorded weekly until the endpoint, defined as either death or sacrifice. Mice were observed for a period of four weeks. Echocardiography was performed at weeks 2, 3, and 4 following BAPN administration. Aortic dissection was defined by the formation of a false lumen containing blood within the medial layer, while aortic aneurysm was classified as arterial dilation exceeding 50% of the normal diameter. All mice were euthanized using a gas anesthetic administered at up to 4.5% Isoflurane in oxygen, continued until respiratory arrest occurred for more than 60 s. Serum and aortic tissue samples were collected for further analysis.

### Enzyme‐Linked Immunosorbent Assay

Serum concentrations of SBDP145 in patients were quantified using a human SBDP145 ELISA kit (CSB‐EQ028022HU, Cusabio). Inflammatory factor levels in human peripheral blood were assessed using a Cytokine Combination Assay Kit (Immunofluorescence assay) at the Laboratory Center of the First Affiliated Hospital of Soochow University. The ELISA was conducted according to the manufacturer's instructions.

### Calpain Activity

Calpain activity was assessed using the assay kit (ab65308, Abcam) according to the manufacturer's instructions. Aortas were isolated from mice and homogenized in 100 µL of extraction buffer on ice for 3 min. Following centrifugation at 21 000 g for 5 min, the supernatant was collected and standardized to a consistent protein amount (50–200 µg) based on total protein measurement. Each sample was supplemented with 10 µL of 10X reaction buffer and 5 µL of calpain substrate, and subsequently incubated at 37 °C in the dark for 1 h. Measurements were conducted using a plate reader equipped with a 400 nm excitation filter and a 505 nm emission filter.^[^ ^56^ ^]^

### Histology

Aorta tissues were harvested, fixed in 4% paraformaldehyde (PFA), embedded in paraffin, and sectioned into 5 µm slices. Histological evaluation was performed using hematoxylin and eosin (HE) staining, while Verhoeff's van Gieson (EVG) staining was applied to assess the presence of elastic and collagen fibers. Pathological changes were examined microscopically.

### Immunofluorescence Staining

Immunofluorescence staining followed previously described protocols.3 Paraffin‐embedded aortic sections and fixed cells were permeabilized with 0.5% Triton X‐100, followed by blocking with 5% BSA. Primary antibodies were incubated overnight at 4 °C. Subsequently, sections and cells were incubated with fluorescent secondary antibodies for 1 h at room temperature, and nuclei were stained with DAPI. Immunofluorescence was visualized using a confocal microscope (Zeiss LSM 510 Mark 4).

### RNA Sequencing

For bulk RNA sequencing, thoracic aorta tissues were harvested from male mice subjected to BAPN induction and treated with the calpain inhibitor Calpeptin. Experimental groups included: Control (Con, *n* = 5), Calpeptin (*n* = 4), BAPN (*n* = 4), and BAPN‐Calpeptin (*n* = 3). Samples were collected at three time points post‐BAPN administration: 0 days (NC, *n* = 3), 14 days (B14, *n* = 3), and 21 days (B21, *n* = 4). Total RNA was extracted using the Trizol reagent kit (Invitrogen, Carlsbad, CA, USA). The cDNA library construction and sequencing were performed on the Illumina NovaSeq 6000 platform at Gene Denovo Biotechnology Co. (Guangzhou, China). Gene expression levels were quantified by calculating FPKM (fragments per kilobase of transcript per million mapped reads) using RSEM software.^[^ ^57^ ^]^ DEGs were identified using DESeq2 software,^[^ ^58^ ^]^ comparing groups with a fold change ≥ 2 and *p*‐value < 0.05.

Series Test of Cluster (STC) analysis was applied to examine expression trends and cluster genes with similar patterns, using a significance threshold of *p* < 0.05.^[^ ^59^ ^]^

Gene Ontology (GO) enrichment analysis was performed to identify significantly enriched GO terms for DEGs compared to the genomic background, as listed in the Gene Ontology database (http://www.geneontology.org/). Pathway enrichment analysis based on the KEGG database was conducted to explore the biological functions of the genes.^[^ ^60^ ^]^ Additionally, Gene Set Enrichment Analysis (GSEA)^[^ ^61^ ^]^ was used to identify significant differences in KEGG pathway‐related gene sets between groups.

Protein‐protein interaction (PPI) networks were generated using the search tool for retrieval of interacting proteins (STRING) database (https://cn.string‐db.org/).

### Quantitative Real‐Time Polymerase Chain Reaction (qRT‐PCR)

The entire aorta from mice was isolated, and total RNA was extracted using Trizol reagent (Invitrogen, CA, USA). SYBR Green 2 × PCR mix (Novoprotein, China) was used for subsequent analysis, following the manufacturer's instructions. GAPDH was employed as a reference gene for the normalization of mRNA expression levels.

### Cell Culture

Primary Human Aortic Endothelial Cells (HAECs) were obtained from ScienCell Research Laboratories and cultured in Endothelial Cell Medium (ScienCell, 1001). HAECs were stimulated with either 1 µmol recombinant human angiopoietin‐II or 100 nmol Calpeptin (MedChemExpress, New Jersey, USA). Cells treated with an equivalent concentration of DMSO were used as controls.^[^ ^62^ ^]^ Calpeptin, a calpain inhibitor, was administered at a 10 µmol concentration for 1 h before angiopoietin‐II stimulation. HAECs were also infected with lentiviral particles containing human Capn2 according to the protocol provided by Hanbio (Shanghai, China).^[^ ^63^ ^]^

HEK293T cells, sourced from the American Type Culture Collection (ATCC, Manassas, VA, USA), were cultured in high‐glucose DMEM supplemented with 10% FBS. These cells were transfected with capn2 or Itgav plasmids following the manufacturer's guidelines from Hanbio (Shanghai, China).

For the adhesion assay, THP‐1 cells (ATCC), were cultured in the RPMI 1640 medium (Life Technologies, Carlsbad, CA).

### Monocyte Adhesion Assay

HAECs were seeded into 12‐well plates at a density of 1 × 10^4^ cells per well and treated with 1 µm AngII for 24 h. THP‐1 cells were suspended at a concentration of 1.0 × 10^6^ cells mL^−1^ and labeled with 10 µmol DiI (MedChemExpress, New Jersey, USA) for a 30‐min incubation. Labeled THP‐1 cells (1 × 10^5^ cells per well) were then seeded onto the HAEC monolayer and incubated at 37 °C for 30 min. Non‐adherent cells were carefully washed away, and the number of adherent THP‐1 cells was quantified using a fluorescent microscope (FSX100, Olympus).^[^ ^64^ ^]^

### Western Blot Analysis

Aortic tissue lysates from both patients and mice were extracted using radioimmunoprecipitation assay (RIPA) lysis buffer (Beyotime, P0013B), and protein concentrations were quantified using the BCA Protein Assay Kit (Beyotime, P0009). Protein extracts were separated via SDS‐PAGE and transferred onto PVDF membranes (Millipore) through electrophoresis. After blocking with 5% non‐fat milk, membranes were incubated overnight at 4 °C with primary antibodies, followed by incubation with HRP‐conjugated secondary antibodies. Visualization of the blots was performed using a two‐color infrared imaging system (Odyssey, LI‐COR Biosciences, USA), and quantification was carried out using ImageJ software.

### Statistical Analysis

Data analysis was conducted using SPSS (version 26.0) and GraphPad Prism (version 10.0; GraphPad Software). Results are expressed as mean ± SD. The Shapiro–Wilk test was applied to evaluate the normality and homogeneity of variance in the datasets. For group comparisons, a two‐tailed unpaired Student's *t*‐test or Mann–Whitney test was used as appropriate. One‐ or two‐way ANOVA with Sidak's or Tukey's tests was employed for multiple group comparisons. Additionally, survival rates and the incidence of thoracic aortic aneurysm/dissection (TAAD) across groups were compared using the log‐rank (Mantel‐Cox) test, Breslow–Wilcoxon test, and χ^2^ test. A *p*‐value of less than 0.05 was considered statistically significant.

## Conflict of Interest

The authors declare no conflict of interest.

## Author Contributions

X.T., Y.W., H.H., and Y.D. contributed equally to this work. X.T. designed the project, analyzed the data, and wrote the manuscript. Y.W. conducted wet lab experiments and acquired data. H.H. and Y.D. participated in data acquisition and analysis. J.W. and M.L. performed the bioinformatic analysis. K.S. contributed to specimen collection. L.S. and Y.Y. provided valuable suggestions regarding the experiment. Z.Y. engaged in the discussion of the results. Z.S. supervised the project and was responsible for securing funding.

## Supporting information



Supporting Information

Supplemental Table 1

Supplemental Table 2

## Data Availability

The data that support the findings of this study are openly available in ScRNA data can be accessed via GEO under the accession number GSE222318, while the mouse RNA‐seq data are available at https://ngdc.cncb.ac.cn/gsub/ (reference number CRA019274 and CRA023370) at [URL/DOI], reference number 4. These data were derived from the following resources available in the public domain: https://www.10.1093/eurheartj/ehad534, https://ngdc.cncb.ac.cn/gsub/ (reference number CRA019274 and CRA023370).
