# Supplementary material for: Calpain‐2‐Mediated Endothelial Focal Adhesion Disruption in Thoracic Aortic Dissection
Source: Adv Sci (Weinh). 2025 Apr 2;12(25):2501112. doi: 10.1002/advs.202501112 (PMC12224997; doi:10.1002/advs.202501112)
Supplement: Supplementary file 1 — Supporting Information [file ADVS-12-2501112-s001.pdf]

ADVANCED SCIENCE
Open Access

Supporting Information

for *Adv. Sci.*, DOI 10.1002/advs.202501112

Calpain-2-Mediated Endothelial Focal Adhesion Disruption in Thoracic Aortic Dissection

Xiaomei Teng, Yansong Wang, Haoyue Huang, Yinglong Ding, Jun Wang, Meili Liu, Kun Song, Lianbo Shao, You Yu, Ziying Yang and Zhenya Shen**

Supporting Information

Title: Calpain-2-mediated Endothelial Focal Adhesion Disruption in Thoracic Aortic Dissection

Authors

Xiaomei Teng, Yansong Wang, Haoyue Huang，Yinglong Ding, Jun Wang, Meili Liu , Kun Song, Lianbo Shao, You Yu, Ziying Yan, Zhenya Shen**

This PDF file includes:

Supplemental figures: Figure S1 to S11

Supplemental tables: Tables S1， S3 to S11,S13

Supplemental figures and legends

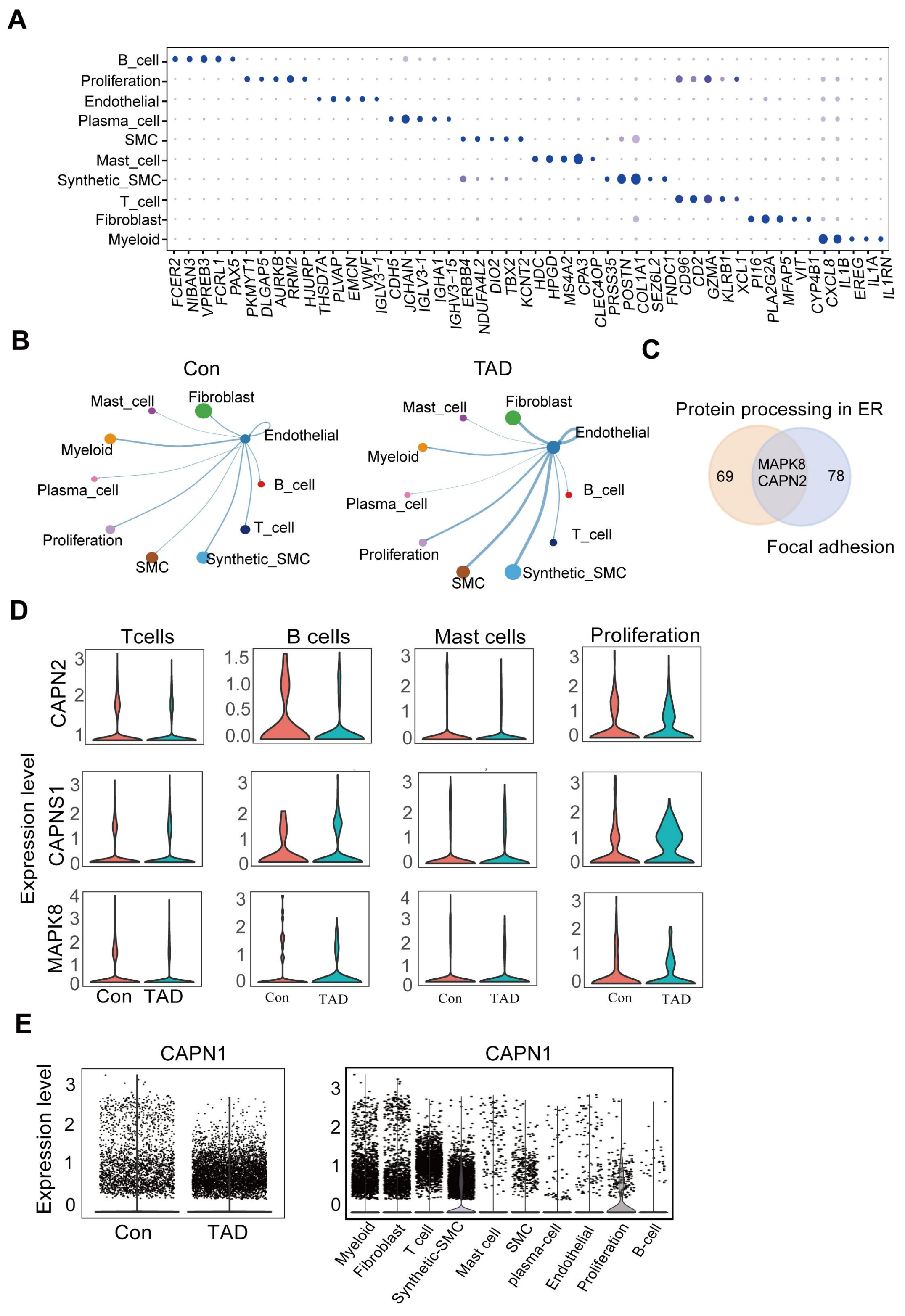

Figure S1. General analysis and annotation of all cells. A) The expression of marker genes was illustrated in the dot plot. B) A network diagram depicted the communication between endothelial cells and other cell types. C) Common genes

identified between two signaling pathways were highlighted. D) A violin plot displayed the expression levels of CAPN2, CAPNS1, and MAPK8 across selected cell types. n=3 pre group

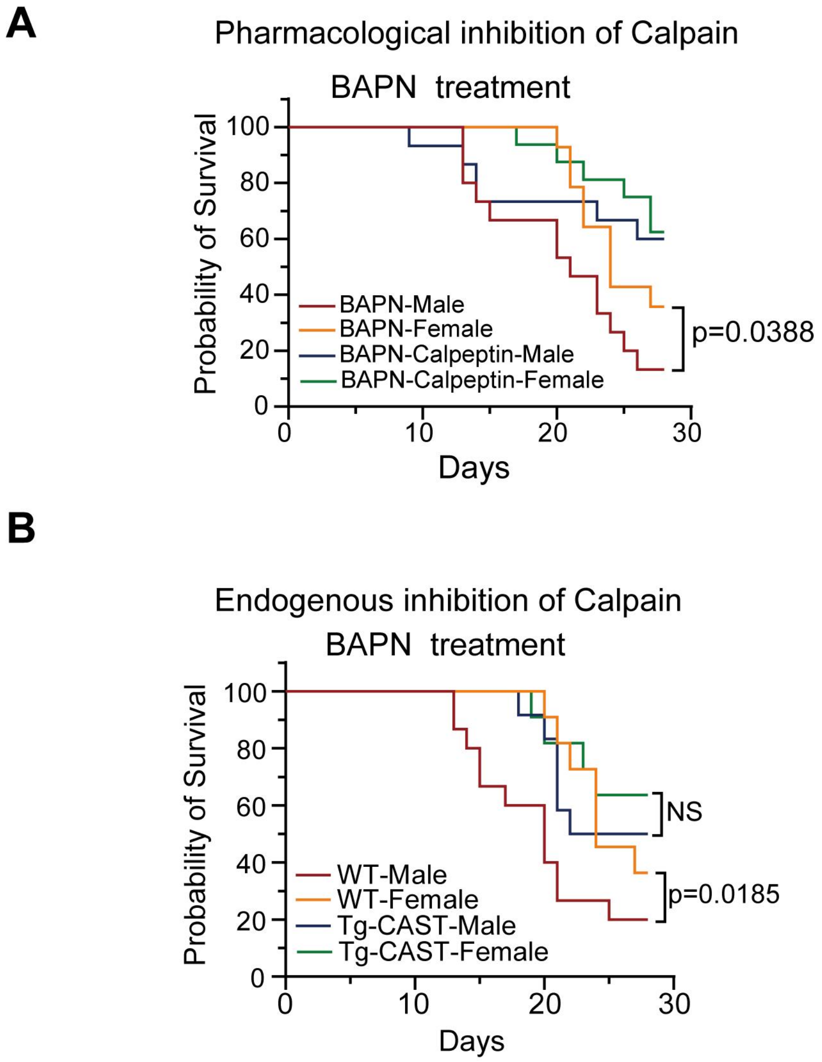

Figure S2. Survival rate of both male and female mice induced by BAPN.

A) Pharmacological inhibition of Calpain. BAPN-male, n = 15; BAPN-Calpeptin-male, n = 15; BAPN-female, n = 14; BAPN-Calpeptin-female, n = 16. B) Over-expressing calpastatin (Tg-CAST) and littermate wild type (WT) mice. WT-male, n = 15; WT-female, n=11; TgCAST-male, n = 12; TgCAST-female, n = 11. The survival rate was estimated using the Kaplan-Meier method and compared via the log-rank test. NS, no significant.

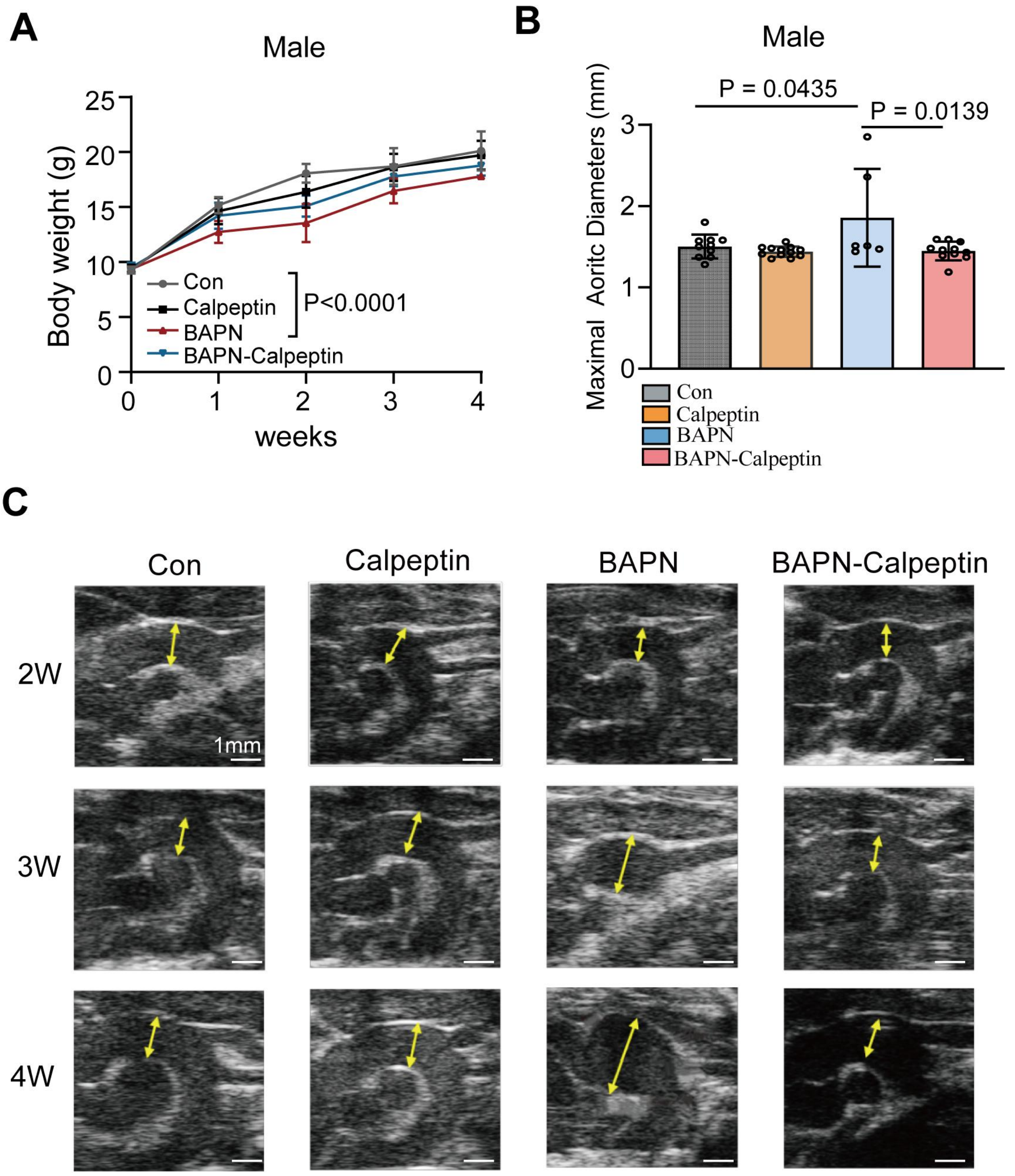

Figure S3. Pharmacological inhibition of Calpain decreased BAPN–induced AD formation in male mice. Con, n=10; Calpeptin, n=12; BAPN, n = 15; BAPN-Calpeptin, n = 15. A) The body weight. B）The maximun aortic diameter among four groups. C) Representative ultrasound images of thoracic aorta. Scale bar: 5mm. Data are presented as the mean ± SD. Statistical significances were determined by two-way ANOVA with Tukey's multiple comparision for (A), and one-way ANOVA with Tukey's multiple comparision for (B).

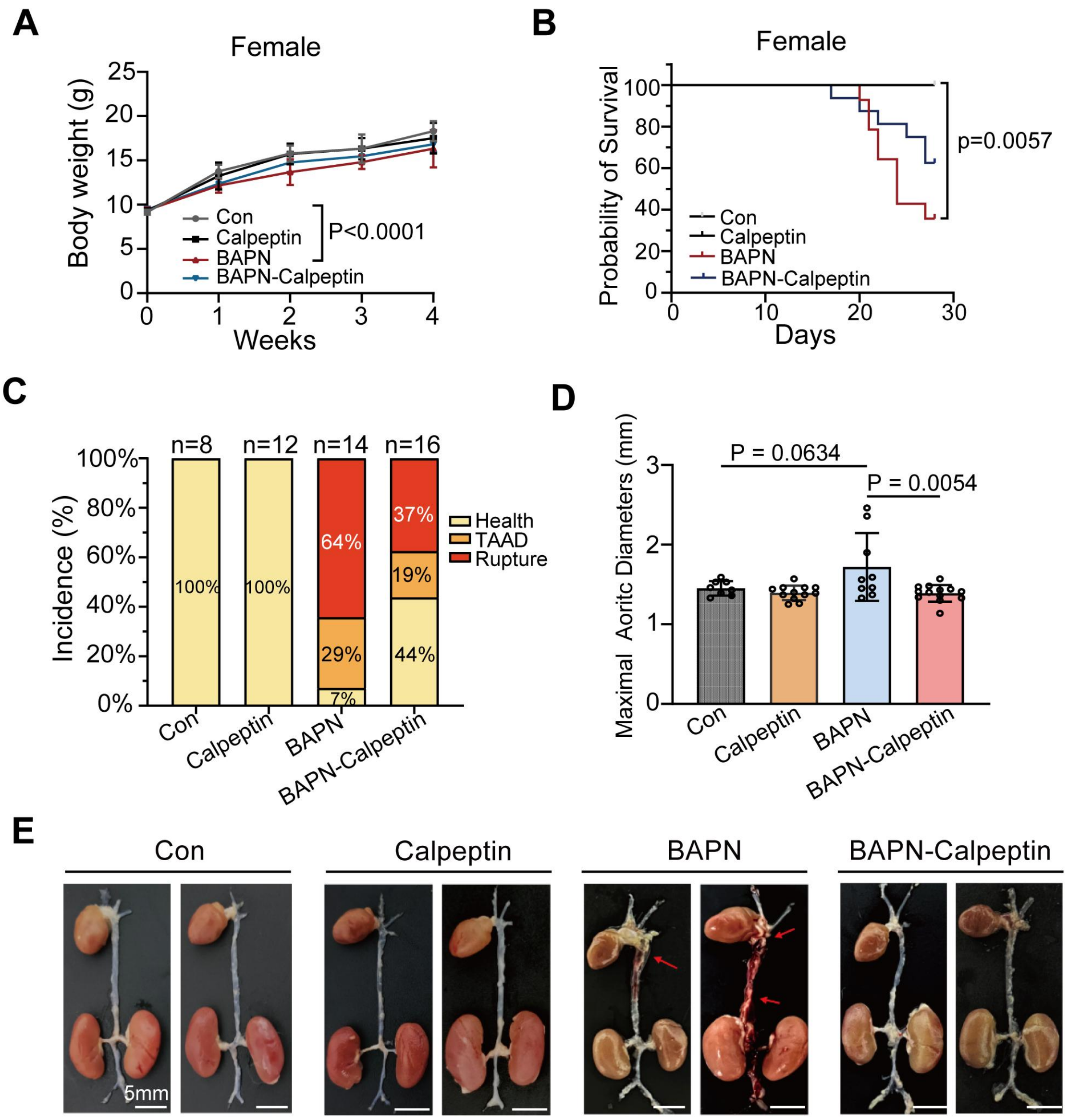

Figure S4. Pharmacological inhibition of Calpain prevented AD formation in female mice. Con, n=8; Calpeptin, n=12; BAPN, n = 14; BAPN-Calpeptin, n = 16. A) The body weight. B）Survival rate was estimated by Kaplan-Meier method and compared by log-rank test. C) TAAD incidence. D) The maximun aortic diameter among four groups. E) Representative macrographs of aorta. Scale bar: 5mm. Data are presented as the mean ± SD. P values were calculated by two-way ANOVA followed by Tukey's multiple comparision in (A), and one-way ANOVA followed by Tukey's multiple comparision in (D).

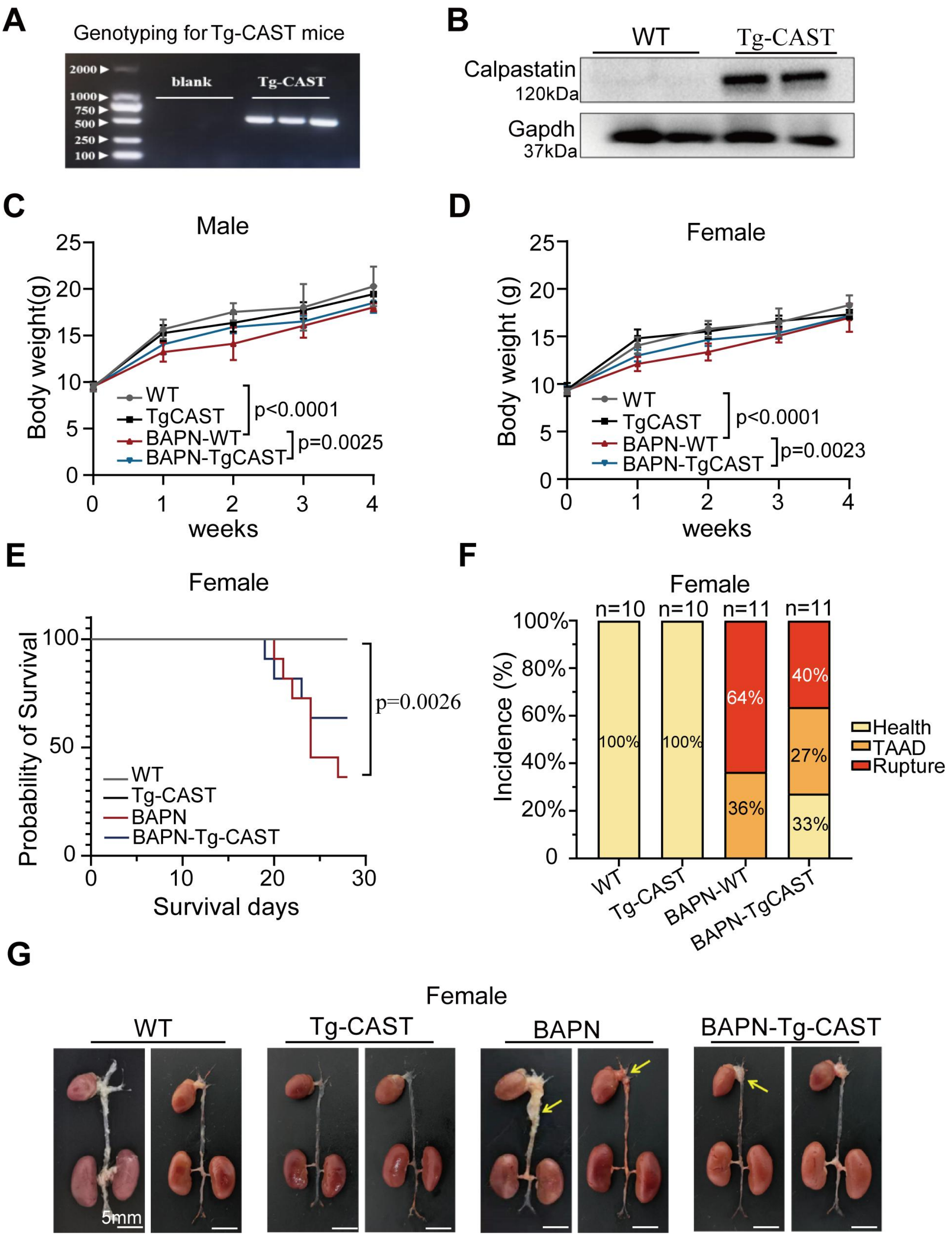

Figure S5. Endogenous inhibition of Calpain in Tg-CAST and littermate WT mice. A and B) Genotyping identification through PCR and western blotting for Calpastatin. C) The body weight of male mice. WT, n=11; Tg-Cast, n=10; BAPN, n = 15; BAPN-TgCAST, n = 12. D) The body weight of female mice. WT, n=10; Tg-Cast, n=10; BAPN, n = 11; BAPN-TgCAST, n = 11. E) Survival rate estimated using the Kaplan-Meier method, with comparisons made via the log-rank test. F) Incidence of TAAD. G) Representative macrographs of the aorta. Scale bar: 5mm. Data are presented as the mean ± SD. P values were calculated by two-way ANOVA, followed by Tukey's multiple comparision in (C) and (D).

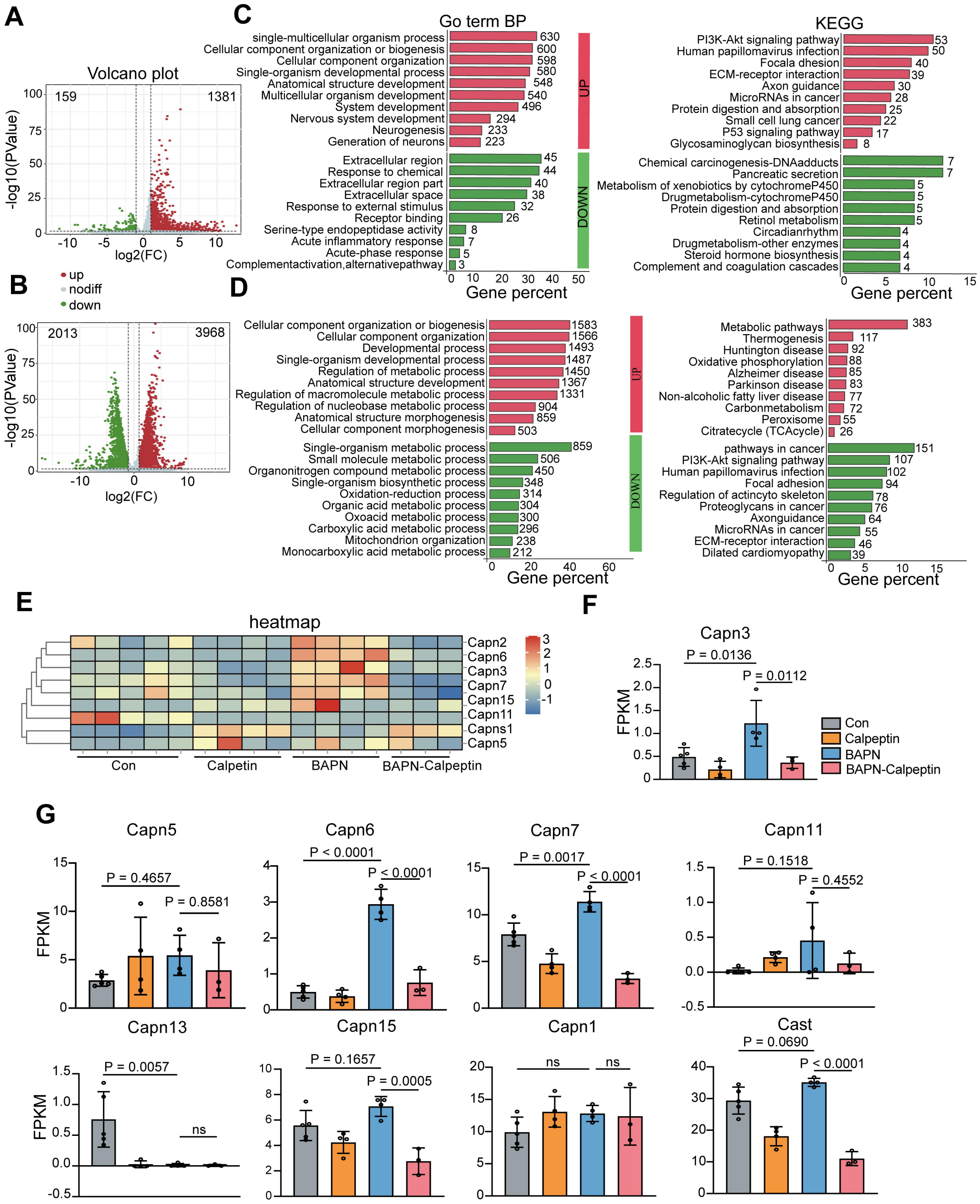

Figure S6. Screening of differentially expressed genes (DEGs) from mice treated with BAPN, with or without Calpeptin. A) Volcano plots of DEGs in aortic tissues from mice with and without BAPN administration. P value < 0.05 and |Log2Fc| > 1. B) Volcano plots of DEGs in aortic tissues from mice induced by BAPN, with or without Calpeptin. C) The top 10 of Gene Ontology (GO) and Kyoto Encyclopedia of Genes and Genomes (KEGG) pathway classifications for up-regulated and down-regulated genes between the control and BAPN-induced groups. P value < 0.05 and |Log2Fc| > 1. D) The top 10 of GO and KEGG pathway classifications for up-regulated and down-regulated genes between the BAPN and BAPN-Calpeptin groups. E) Heatmap of the Calpain family across the four groups. The Fragments Per Kilobase of

transcript per Million mapped reads (FPKM) value of members for Capn3 (F) and other isoforms (G). Con, n = 5; Calpeptin, n = 4; BAPN, n = 4; BAPN-Calpeptin, n = 3. Data are presented as the mean ± SD. Statistical significances were determined by one-way ANOVA with Tukey's multiple comparision.

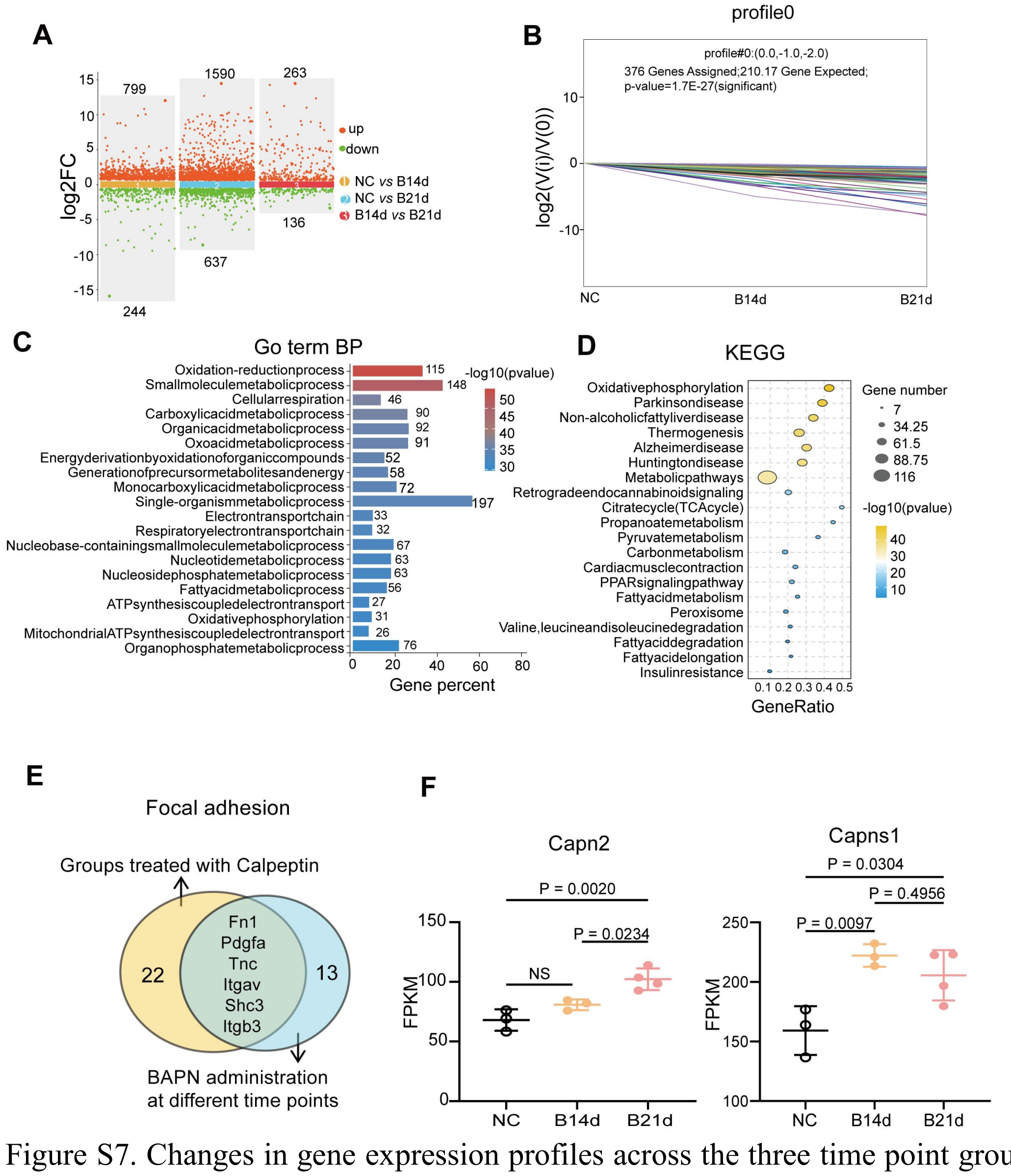

Figure S7. Changes in gene expression profiles across the three time point groups. A) The number of up-regulated and down-regulated genes between groups (|log2FC| > 1, P values < 0.05). B) The expression trend of genes in profile 0. C) The top 20 enrichment analyses of the KEGG pathway. D) The top 20 enrichment analyses of GO terms in biological processes. E) The common genes associated with the focal adhesion pathway identified between the two RNA-seq datasets. F) FPKM values of Capn2 and Capns1 across the three groups. NC, n = 3; B14d, n = 3; B21d, n = 4. Data are presented as the mean ± SD. Statistical significances were determined by one-way ANOVA with Tukey's multiple comparision.

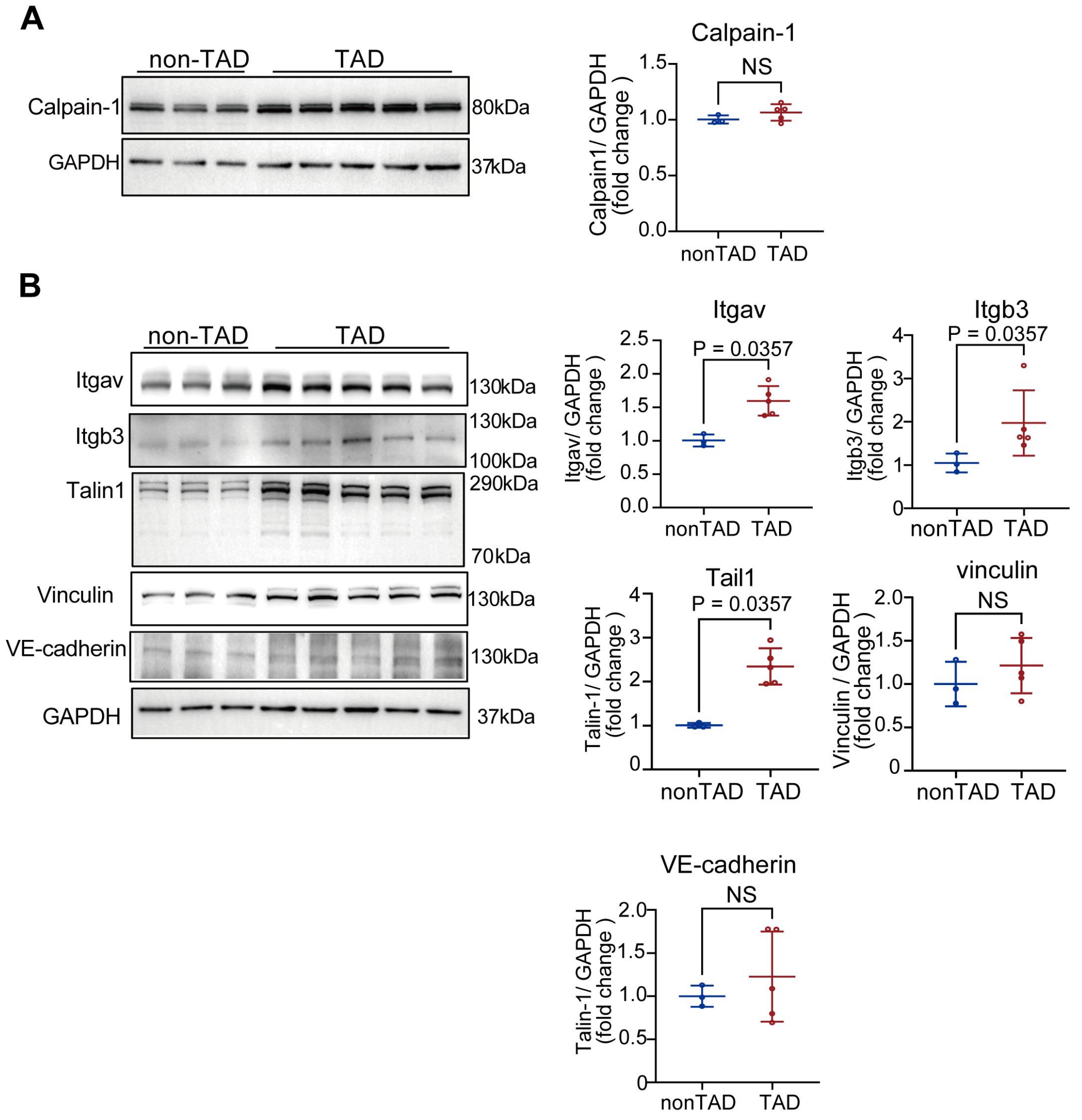

Figure S8. The expression of proteins in aortic samples. The expression levels of Calpain-1, Itgav, Itgb3, Talin1, Vinculin, and VE-cadherin were assessed using Western blotting. Representative Western blot images in aortic specimens were from non-TAD (n=3) and patients with TAD (n=5). Data are presented as the mean ± SD. Statistical significances were determined by One-way ANOVA with Tukey's multiple comparision.

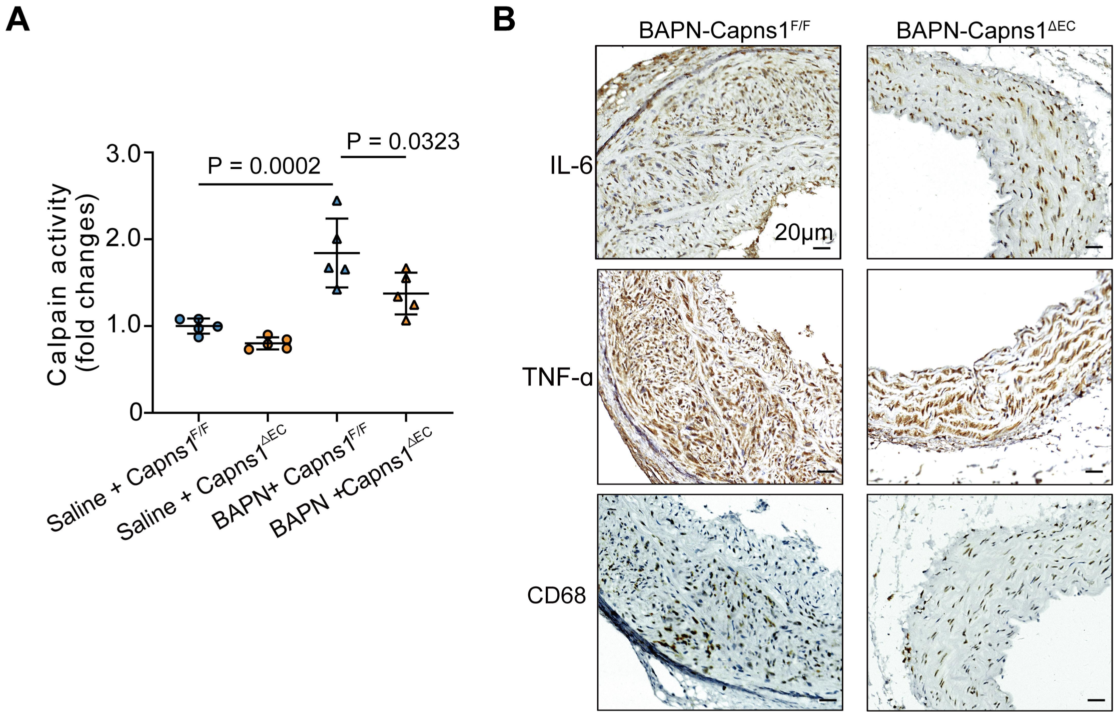

Figure S9. Conditional knockout of capnsl in endothelial cells reduced the inflammation of BAPN-induced mice. A) Calpain activity was estimated using the assay kit. n = 5 per group. Data are presented as the mean ± SD. Statistical significances were determined by One-way ANOVA with Tukey's multiple comparision. B) Representative image of immunohistochemical staining for IL-6, TNF-α and CD68^{+} macrophages. Scale bar, 20μm.

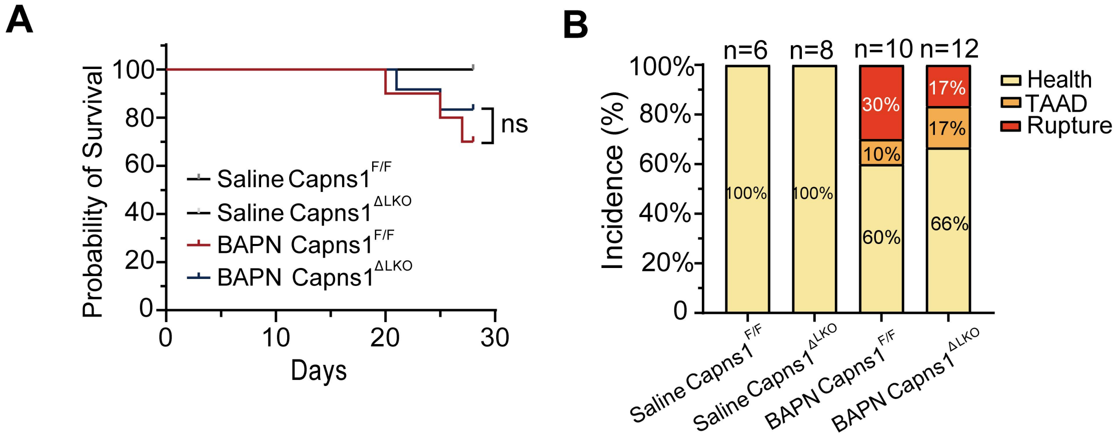

Figure S10. Conditional knockout of capnsl in macrophages reduced the ruputure of BAPN-induced TAAD. A) The survival rate was estimated using the Kaplan-Meier method and compared via the log-rank test. B) The incidence of TAAD in Capns1$^{F/F}$ and Capns1$^{\triangle LKO}$ mice following 4 weeks of BAPN treatment. Saline-Capns1$^{F/F}$, n = 6; Saline-Capns1$^{\triangle LKO}$, n = 8; BAPN-Capns1$^{F/F}$, n =10; BAPN-Capns1$^{\triangle LKO}$, n = 12.

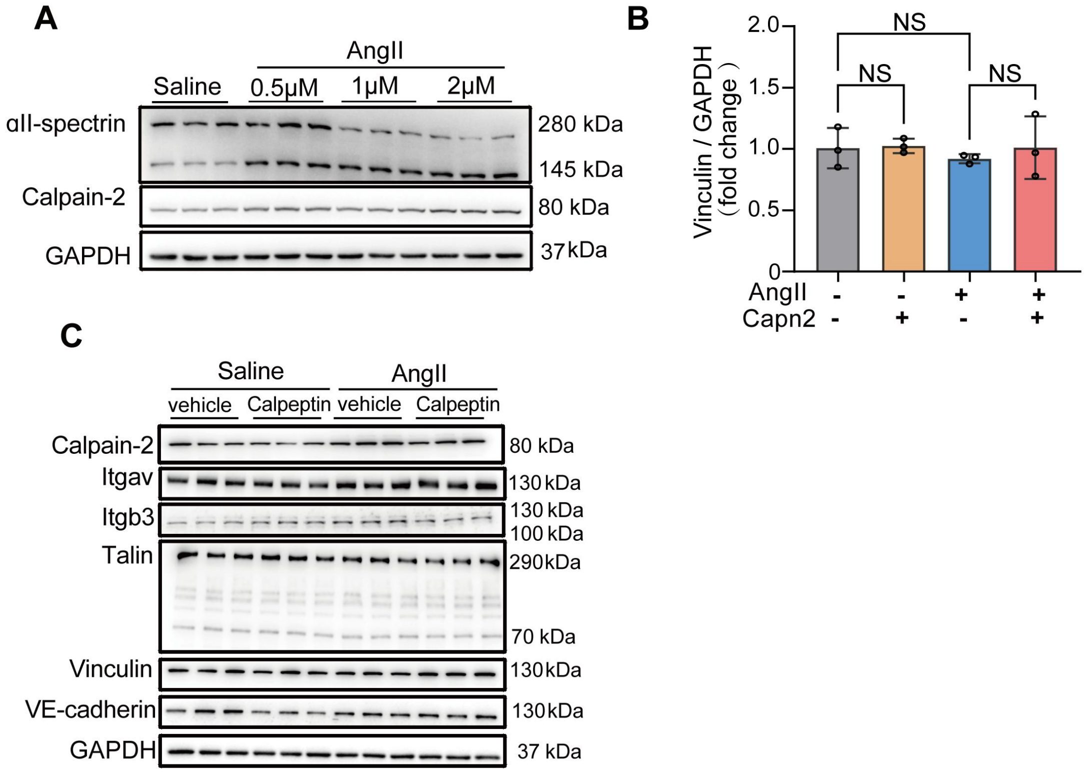

Figure S11. The role of Calpain-2 in endothelial cells. A) HAECs were treated with 0.5, 1 and 2 μmol Angiotensin II (Ang II) for 24 hours. Calpain activity(145 fragment of αII-spectrin) and Calpain-2 were measured by WB. B) Quantitative data from the Western blot analysis for Vinculin are presented. n=3 Data are presented as the mean ± SD. Statistical significances were determined by One-way ANOVA with Tukey's multiple comparision. C) HAECs were treated with 1 μmol Angiotensin II (Ang II) for 24 hours. The calpain inhibitor Calpeptin was administered at a concentration of 10 μmol for 1 hour prior to the Ang II treatment. Representative Western blot images for Calpain-2, Itgav, Itgb3, Talin1, Vinculin, and VE-cadherin are shown, with GAPDH serving as the loading control.

Supplementary tables

Table S1. Data characteristics of the control group and TAD group

Cell	Control		TAD	
	types number of cells	ratio(%)	types number of cells	ratio(%)
Myeloid	5211	24.16	7687	29.07
Fibroblast	7004	32.47	171	0.65
T cell	5158	23.91	9436	35.68
B cell	48	0.22	174	0.66
SMC	281	1.30	1290	4.88
Synthetic_SMC	471	2.18	5409	20.45
Endothelial	911	4.22	249	0.94
Proliferation	188	0.87	321	1.21
Plasma cell	771	3.57	646	2.44
Mast cell	1530	7.09	1064	4.02
Total	21573	100	26447	100

Control data from GSM6919843, GSM6919844, GSM6919845, n=3; TAD data from included GSM6919837, GSM6919839, GSM6919840, n=3.

Tables S3. Clinical characteristics of the Patients with TAD

Variable	TAD
Number, n	25
Gender (male), n (%)	19 (76.00%)
Age, years	53.96 ± 2.702
BMI, kg/m^2	27.12 ± 0.81
Comorbidity, n (%)	
Hypertension	20 (80.00%)
Diabetes	1 (4.00%)
Coronary artery disease	2 (8.00%)
COPD	0 (0.00%)
Chronic kidney disease	0 (0.00%)
Smoke	4(16.00%)

Data are presented as n (%) for categorical data, mean ± SD for continuous data. BMI, body mass index. COPD, Chronic Obstructive Pulmonary Disease.

Table S4. Laboratory characteristics of the Patients with TAD

Variable	Value	Reference range
Blood routine index		
WBC count, 10^9/L	12.18 ± 0.90	3.50-9.50
Neutrophil count, 10^9/L	10.40 ± 0.84	1.80-6.30
Lymphocyte count, 10^9/L	1.05 ± 0.10	1.10-3.20
Monocyte count, 10^9/L	1.17 ± 0.34	0.10-0.60
Hemoglobin, g/L	127.12 ± 4.54	115-150
Platelet count, 10^9/L	185.00 ± 11.08	125-350
RDW, %	13.62 ± 0.35	8.7-18.1
CRP, mg/L	34.89 ±12.50	0-4
Comprehensive Metabolic Panel		
TBil, μmol/L	22.780 ± 2.47	⩽23.0
ALT, U/L	69.852 ± 30.06	9-50
AST, U/L	89.136 ± 43.54	15-40
Albumin, g/L	38.84 ± 0.90	40-55
Creatinine, μmol/L	115.84 ± 23.28	50-90
Cystatin C, mg/L	1.12 ± 0.11	0.59-1.03
Blood glucose, mmol/L	7.04 ± 0.40	3.9-6.1
TC, mmol/L	4.23 ± 0.23	＜5. 2
TG, mmol/L	1.21 ± 0.12	＜1. 7
HDL-C, mmol/L	1.01 ± 0.06	⩾1.0
LDL-C, mmol/L	2.56 ± 0.20	＜3. 4
Ca, mmol/L	2.14 ± 0.02	2.11-2.52
K, mmol/L	3.80 ± 0.17	3.5-5.3
Na, mmol/L	140.10 ± 0.48	137-147
P, mmol/L	1.38 ± 0.15	0.85-1.51
Coagulation function		
PT, s	14.66 ± 0.42	11.5-15.5
APTT, s	37.68 ± 1.27	26-40
D-Dimer, μg/ml	9.21 ± 1.56	0-0.5
Chest Pain Set		
hs-cTnT, pg/ml	49.47 ±23.89	0-14
Myo, ng/ml	203.42 ± 119.54	＜72
CK-MB, ng/ml	6.27 ± 2.50	0-125
NT-proBNP, pg/mL	427.24 ± 120.07	0-4.87

Data are expressed as mean ± SD. n=25. WBC, white blood cell; RDW：red cell distribution width; CRP, C-reactive protein; TBil：Total Bilirubine Tbil; ALT：alanine aminotransferas; AST：aspartate transaminase; TC：Total cholesterol；TG：triglyceride; HDL-C：high-density lipoproteincholesterol; LDL-C：low-density lipoprotein cholesterol; PT：prothrombin time; APTT：activated partial thromboplastin time; Myo: myohemoglobin; hs-cTnT：Hypersensitive troponin T; CK-MB：creatine kinase MB.

Table S5. Laboratory characteristics of the Patients with TAD

Variable	Value	Reference range
IL-2, pg/ml	1.29 ± 0.09	⩽7.5
IL-4, pg/ml	2.21 ± 0.22	⩽8.56
IL-5, pg/ml	1.00 ± 0.09	⩽3.1
IL-6, pg/ml	389.38 ± 120.28	⩽5.4
IL-8, pg/ml	40.46 ± 16.53	⩽20.6
IL-10, pg/ml	47.20 ±20.60	⩽12.9
TNF-α, pg/ml	3.26 ± 0.50	⩽16.5
IFN-γ, pg/ml	2.49 ± 0.20	⩽23.1
IL-17a, pg/ml	8.04 ± 1.24	⩽21.4
IL-1β, pg/ml	2.23 ± 0.21	⩽12.4
IL-12P70, pg/ml	3.48 ± 0.41	⩽3.4
IFN-γ, pg/ml	2.95 ± 0.34	⩽23.1

Data are expressed as mean ± SD. n=25

Table S6. Characteristics of the Patients with TAD and Control group

	TAD	Control	P value
Number, n	25	25	
Gender (male), n (%)	19 (76.00%)	19 (76.00%)	1.000
Age, years	53.96 ± 2.70	53.07 ± 2.69	0.973
Serum SBDP145, pg/ml	1584.06 ± 216.39	818.32 ± 118.78	0.0032

Data are presented as n (%) for categorical data, mean ± SD for continuous data. P values are for comparisons between controls and all patients (chi-square test of homogeneity was used for categorical variables and two-tailed unpaired t-test for continuous variables).

Table S7. Characteristics of the Patients with TAD and Control group

Organ Function Impairment	SBDP145 ≥1207.10 pg/ml, n = 15	SBDP145 ＜1207.10 pg/ml, n = 10
Kidney injury		
No	11 (73.33%)	10 (100.00%)
Yes	4 (26.67%)	0 (0.00%)
Liver injury		
No	12 (80.00%)	9 (90.00%)
Yes	3 (20.00%)	1 (10.00%)
Myocardial injury		
No	12 (80.00%)	10 (100.00%)
Yes	3 (20.00%)	0 (0.00%)

Data are presented as n (%) for categorical data.

Table S8. Mean arterial diameter of male mice

Vascular (mm)	Control n=10	Calpeptin n=12	BAPN n=15	BAPN+ Calpeptin n=15	P value
Aortic root	1.62±0.07	1.64±0.06	1.69±0.02	1.66±0.02	0.884
Arcus aortae	1.50±0.05	1.44±0.02	1.86±0.25	1.45±0.03	0.010
Descending aortae	1.14±0.04	1.19±0.03	1.32±0.06	1.20±0.04	0.093
Abdominal Aorta	0.95±0.03	0.88±0.05	1.00±0.03	0.93±0.03	0.314
Brachiocephalic trunk	0.77±0.01	0.77±0.02	0.83±0.05	0.74±0.03	0.131
Left common carotid artery	0.58±0.02	0.55±0.02	0.59±0.02	0.56±0.01	0.552
Left subclavian artery	0.54±0.02	0.50±0.02	0.56±0.02	0.53±0.01	0.192

Data are expressed as mean ± SD. Statistical significances were determined by one-way ANOVA with Tukey's multiple comparision.

Table S9. Mean arterial diameter of female mice

Vascular (mm)	Control n=8	Calpeptin n=12	BAPN n=14	BAPN+ Calpeptin n=16	P value
Aortic root	1.68±0.06	1.68±0.03	1.69±0.05	1.64±0.05	0.880
Arcus aortae	1.45±0.03	1.39±0.03	1.72±0.14	1.39±0.03	0.004
Descending aortae	1.08±0.02	1.17±0.04	1.21±0.02	1.18±0.07	0.350
Abdominal Aorta	0.91±0.04	0.87±0.03	0.94±0.04	0.93±0.03	0.358
Brachiocephalic trunk	0.66±0.04	0.69±0.02	0.71±0.02	0.69±0.02	0.672
Left common carotid artery	0.53±0.02	0.50±0.02	0.54±0.03	0.52±0.01	0.435
Left subclavian artery	0.47±0.03	0.43±0.02	0.50±0.02	0.49±0.02	0.149

Data are expressed as mean ± SD. Statistical significances were determined by one-way ANOVA with Tukey’s multiple comparision.

Table S10. Mean arterial diameter of male mice

Vascular（mm）	WT<br>n=11	Tg-CAST<br>n=10	BAPN-WT<br>n=15	BAPN-Tg-CAST<br>n=12	P<br>value
Aortic root	1.48±0.04	1.60±0.04	1.63±0.09	1.58±0.04	0.173
Arcus aortae	1.44±0.04	1.44±0.05	1.68±0.28	1.55±0.07	0.421
Descending aortae	1.09±0.04	1.21±0.04	1.31±0.06	1.28±0.05	0.008
Abdominal Aorta	0.83±0.02	0.90±0.03	0.92±0.06	0.90±0.04	0.202
Brachiocephalic trunk	0.68±0.03	0.65±0.02	0.72±0.06	0.67±0.03	0.523
Left common carotid artery	0.53±0.01	0.56±0.01	0.58±0.04	0.55±0.02	0.337
Left subclavian artery	0.52±0.01	0.50±0.01	0.54±0.03	0.50±0.02	0.482

Data are expressed as mean ± SD. Statistical significances were determined by one-way ANOVA with Tukey’s multiple comparision.

Table S11. Mean arterial diameter of female mice

Vascular（mm）	WT n=10	Tg-CAST n=10	BAPN-WT n=11	BAPN-Tg-CAST n=11	P value
Aortic root	1.60±0.06	1.48±0.06	1.69±0.03	1.66±0.09	0.170
Arcus aortae	1.48±0.04	1.28±0.04	1.66±0.10	1.46±0.03	0.002
Descending aortae	1.10±0.02	1.15±0.05	1.33±0.10	1.16±0.04	0.027
Abdominal Aorta	0.91±0.03	0.88±0.03	0.92±0.06	0.90±0.04	0.895
Brachiocephalic trunk	0.67±0.04	0.66±0.03	0.70±0.02	0.68±0.02	0.885
Left common carotid artery	0.55±0.02	0.49±0.02	0.56±0.02	0.51±0.02	0.035
Left subclavian artery	0.51±0.03	0.49±0.02	0.53±0.03	0.52±0.01	0.667

Data are expressed as mean ± SD. Statistical significances were determined by one-way ANOVA with Tukey’s multiple comparision.

Table S13: Primer sets used in the present study (Mus, Mouse)

Gene names	Forward (5'-3')	Reverse (5'-3')	Application
Mus Gapdh	AGGTCGGTGTGAACGGATTTG	TGTAGACCATGTAGTTGAGGTCA	RT-qPCR
Mus Capn1	ATGACAGAGGAGTTAATCACCCC	GGCTATGAGAAACCGGAGGG	RT-qPCR
Mus Capn2	GGTCGCATGAGAGAGCCATC	CCCCGAGTTTTGCTGGAGTA	RT-qPCR
Mus Capn3	AACACAGTGGTGAACAAACACA	ACTGCATTTCGCATCTCATAGC	RT-qPCR
Mus Capns1	ATCCTGGGCGGAGTCATTAGT	TTTCCTGAACTGACGGACCTC	RT-qPCR
Mus Capn5	CACCGACGACTCCCTTTACTA	GAGCTGATGCCATCTACGAAG	RT-qPCR
Mus Capn6	GGAAGCGTCCACAGGACATTT	GTGGTTGGGAATTGCCTTTGT	RT-qPCR
Mus Capn7	GAAGGCCGCTACTCTGAGG	GGGCTTGCACTCTTTCCAGAT	RT-qPCR
Mus Itgav	CCGTGGACTTCTTCGAGCC	CTGTTGAATCAAACTCAATGGGC	RT-qPCR
Mus Vcl	GAGGCTGAACTGCTTCAATCA	CCAGATTTGACGAGGTGCCTA	RT-qPCR
Mus Tln1	CCTGCCGCATGATTCGTGA	TCGGAGCATGTAGTAGTCCAAA	RT-qPCR
Mus Cre	GTTGGCTTAGGCTGCTTTTCGT	CCAGACTCCGTGACACCCCTT	PCR
